# Electrostatic and Structural Engineering in Preintercalated V_2_O_5_: A Design Framework for Multivalent Ion Storage

**DOI:** 10.1002/smsc.70338

**Published:** 2026-07-28

**Authors:** Anagha P. Vincent, P. Vipin Kumar, S. B. Gudennavar, B. S. Nishchith, S. G. Bubbly, Maria Helena Braga

**Affiliations:** ^1^ Department of Physics and Electronics CHRIST University Bengaluru India; ^2^ CoE Battery Engineering Atria University Bangalore India; ^3^ LAETA Faculty of Engineering University of Porto Porto Portugal; ^4^ MatER Faculty of Engineering University of Porto Porto Portugal

**Keywords:** interlayer spacing, metal‐ion intercalation, vanadium pentoxide (V_2_O_5_), zinc‐ion battery

## Abstract

Preintercalation has emerged as a powerful strategy to modulate the electrochemical performance of layered vanadium oxides, yet a unified mechanistic understanding linking structural and electrostatic effects remains lacking. Here, we establish a unified framework describing how preintercalated species govern ion transport, structural stability, and electrochemical behavior in V_2_O_5_‐based cathodes. We show that preintercalation operates through a coupled structural–electrostatic mechanism governed by interlayer spacing, charge screening, and solvation effects, where guest species act as interlayer pillars that stabilize the lattice while simultaneously tuning the local electrostatic potential landscape and charge screening environment. The interplay between ionic size, charge density, hydration state, and electronic structure defines Zn^2+^ diffusion barriers, redox kinetics, and electrochemical reversibility. By systematically analyzing monovalent, divalent, and trivalent preintercalated metal ions, we identify key design principles that dictate optimal interlayer spacing, electronic conductivity, and ion mobility. Importantly, the role of structural water is redefined as an active electrostatic mediator reducing effective ion charge and lowering migration barriers. This work transcends conventional material‐specific discussions and provides a generalizable design framework for preintercalation engineering in layered oxides, with implications extending beyond zinc‐ion systems to multivalent and hybrid energy storage technologies.

## Graphical abstract

Preintercalation is presented as a coupled structural–electrostatic strategy for regulating ion storage in layered V_2_O_5_ cathodes. Interlayer spacing, guest‐ion charge density, structural water, migration barriers, and electronic structure are integrated into a unified design framework that clarifies how preintercalated species stabilize the lattice, accelerate Zn^2+^ transport, and improve electrochemical reversibility.

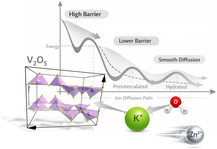

## 1 Introduction

Zinc‐ion batteries (ZIBs) are being developed as safer, low‐cost, and environmentally friendly alternatives to lithium‐ion batteries. They offer a high theoretical capacity of 820 mAh g^−1^, along with several key advantages, including the use of nonflammable aqueous electrolytes, abundant, inexpensive, nontoxic, and recyclable zinc [1, 2, 3, 4, 5, 6, 7]. A typical ZIBs consists of a zinc metal anode, a zinc‐ion hosting cathode, and a zinc‐containing electrolyte. While discharging, the ions migrate through the electrolyte to the cathode, where they are intercalated [8, 9]. This process is reversed during charging.

ZIBs typically operate within a voltage range of 1.0–1.8 V, have an energy density of 100–200 Wh kg^−1^, a cycle life of 500–3000 cycles, and a Coulombic efficiency of ∼95% [10], but remain limited by sluggish multivalent ion diffusion and interfacial instability. Zinc shows good stability in aqueous electrolytes like ZnSO_4_ and Zn(CF_3_SO_3_)_2_. However, Zn‐ion batteries face challenges, including dendrite formation caused by Zn plating during cycling, which can cause short circuits, cathode degradation resulting from repeated Zn^2+^ insertion, and hydrogen evolution due to water decomposition at low potentials, which limits their voltage window and energy density compared to Li‐ion batteries [3, 11, 12].

Over the years, various cathode materials have been explored to address the sluggish Zn^2+^ insertion/extraction kinetics observed in traditional cathodes. These include metal oxides, polyanionic frameworks, Prussian blue analogs, layered sulfides, organic cathode hosts, conductive polymers, and MXene‐based materials among others [13]. ZIBs generally operate in aqueous electrolytes, hence chemical stability and redox compatibility are of prime importance. In this context, metal oxides have emerged as a suitable and extensively explored class of cathode materials [14]. Many conventional cathode materials, such as layered sulfides and organic frameworks, undergo rapid structural degradation or dissolution in aqueous zinc electrolytes due to strong electrostatic interactions and structural instability under hydrated Zn^2+^ insertion. Metal oxides possess strong metal—oxygen bonds, which contribute to enhanced chemical and structural stability, as well as to reversible Zn^2+^ redox reactions. Furthermore, metal oxides can be tuned to layered, tunneled, and open‐framework architectures that accommodate even relatively large, hydrated Zn^2+^ ions with negligible volume change during cycling. Some of the prominent metal oxide cathodes are MnO_2_, V_2_O_5_, VO_2_, V_6_O_13_, V_2_O_5_⋅nH_2_O, MoO_3_, TiO_2_, etc. [15, 16, 17, 18, 19, 20, 21]. Manganese oxides and Prussian blue analogs are also extensively explored, but these materials often suffer from limited specific capacity and/or poor cycling stability [9, 22, 23].

**FIGURE 1:**
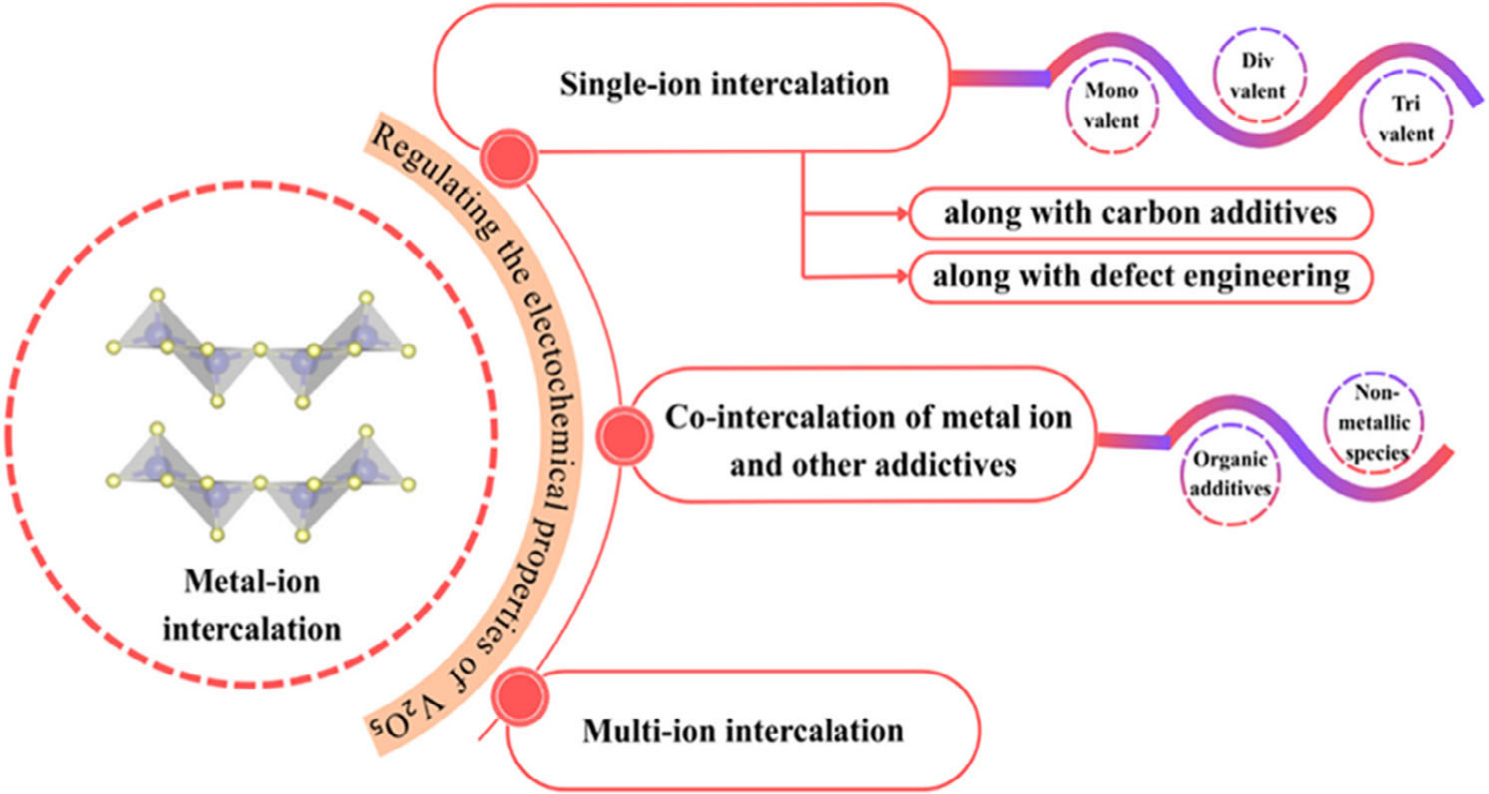
Schematics highlighting the options to regulate the electrochemical properties of V_2_O_5_. Original to this study.

In contrast, vanadium‐based metal oxides gain attention among the metal oxides, as they have very high capacity, structural diversity, and natural abundance [24]. However, the fundamental design rules governing their stability and ion transport remain unclear. Vanadium‐based metal oxides also have multiple oxidation states, such as V^5+^, V^4+^, and V^3+^, forming layered or open structures where Zn^2+^ intercalation can occur. Among them, vanadium pentoxide (V_2_O_5_) stands out as the most widely used cathode in ZIBs, owing to its high theoretical capacity of 589 mAh g^−1^, stable layered structure, long cycle life, low cost, wide availability and high reversibility in Zn^2+^ intercalation/deintercalation [25, 26]. Due to its intrinsic properties, one can modify and optimize metal‐ion intercalation and structural engineering of V_2_O_5_ more easily than other compounds of vanadium [27, 28, 29] (Figure 1). Though V_2_O_5_ has a significant advantage over other metal oxides, the structural degradation caused by the collapse of the interlayer spacing over repeated cycles is a major challenge to be addressed [30].

**FIGURE 2:**
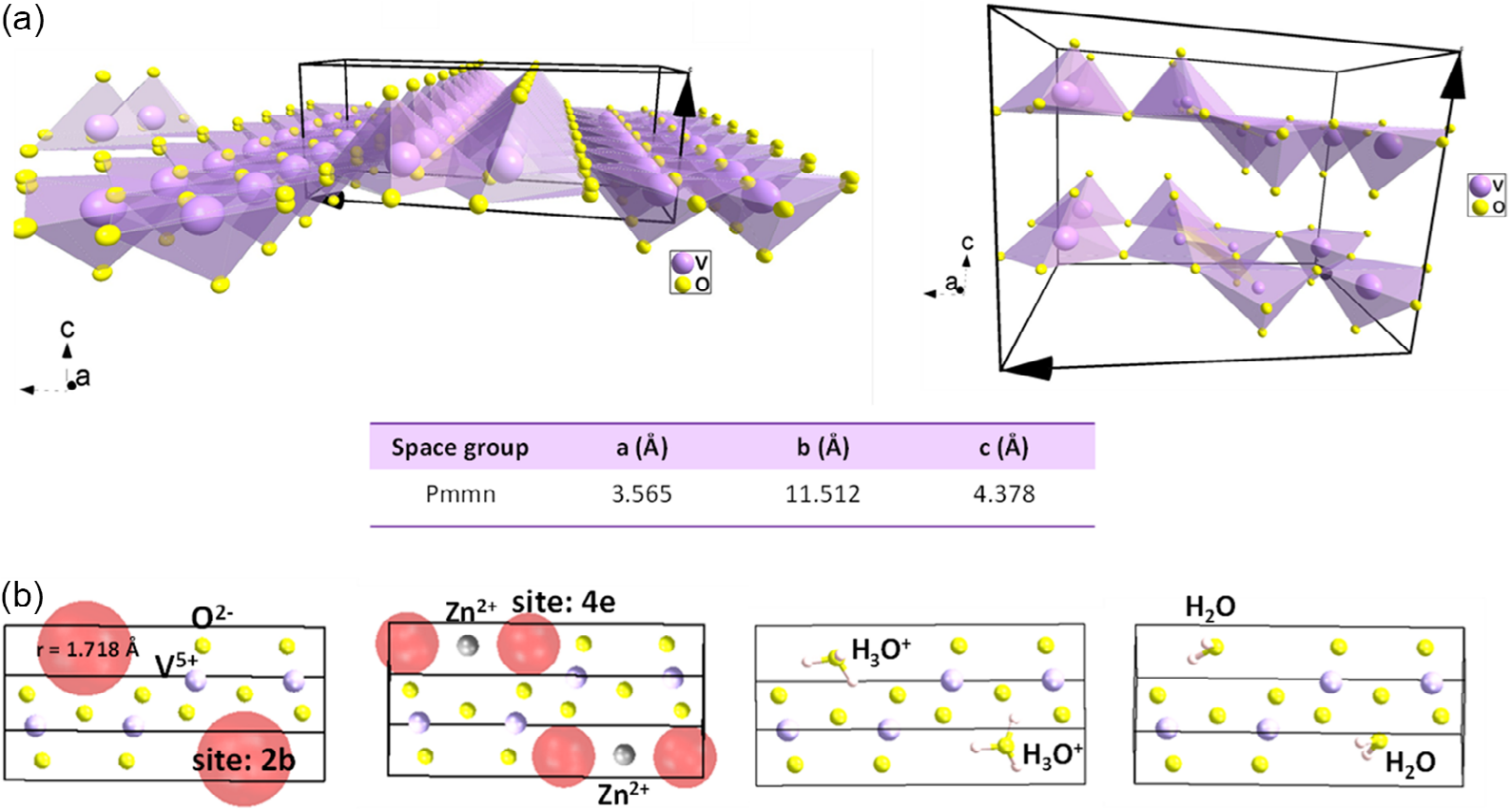
Crystal structures highlighting: (a) V_2_O_5_ orthorhombic (s.g.: Pmmn). (b) Examples of possible V_2_O_5_ intercalated species Zn^2+^, H_2_O, and H_3_O^+^ onto the layers of V_2_O_5_ framework. Original to this study.

Electrochemical performance of V_2_O_5_ depends heavily on its lattice/layer structure, surface states, and redox behavior. Repeated Zn^2+^ insertion/extraction can distort the VO_5_ pyramids in V_2_O_5_ and cause structural degradation, often becoming a key factor limiting battery lifespan and performance [31, 32]. Several strategies have been explored to improve the structural stability and optimize the local electron/charge distribution and electrochemical redox performance [33, 34]. Strategies like defect engineering, preintercalation, structural engineering, nanostructuring, carbon‐based composites, polymer or organic treatments, surface modifications, etc. are being explored [16, 35, 36]. Preintercalation stabilizes V_2_O_5_ through interlayer pillaring and electrostatic screening, mitigating lattice collapse during Zn^2+^ insertion [37]. A wide range of monovalent, divalent, and trivalent metal ions have been explored as intercalants within the V_2_O_5_ framework to enhance electrochemical properties [38] (Figure 2). Such preintercalated metal ions enhance structural stability through strong host–guest interactions, intrinsic conductivity due to excess electrons, and reduces vanadium dissolution while acting as a strong pillar for Zn^2+^ ions to intercalate. This also enables faster Zn^2+^ diffusion and greater stability in ZIBs. Optimal intercalants balance ionic size, charge density, and chemical stability to maximize interlayer spacing while preserving Zn^2+^ mobility (Figure 2). Optimal intercalants exhibit a balance of ionic size, charge density, and chemical stability in aqueous electrolytes.

This review highlights monovalent, divalent, and trivalent metal‐ion intercalation strategies within the V_2_O_5_ framework. In hydrated V_2_O_5_, the structural water affects the interlayer spacing (Figure 2b), thereby influencing ion diffusion and intercalation.

### 1.1 Role of Structural Water in Bilayer V_2_O_5_

Bilayer‐structured V_2_O_5_⋅nH_2_O (VOH) is emerging as a promising cathode material for aqueous ZIBs. Structural H_2_O modulates interlayer electrostatics and solvation dynamics, driving structural changes and tuning the performance of vanadium oxides. Their involvement is normally inescapable during hydrothermal synthesis and when aqueous electrolytes are employed. Structural water between V_2_O_5_ sheets increases interlayer spacing and shields charge interactions during Zn^2+^ intercalation, thus decreasing the binding energy between Zn and V_2_O_5_, as supported by density functional theory (DFT) calculations and electrochemical measurements [39] (Figure 2b). Moreover, oxygen atoms in H_2_O can serve as additional host sites to accept electrons from zinc, leading to an increased open‐circuit voltage. Meanwhile, structural water creates a more uniform electrostatic environment, thereby lowering the diffusion barrier for Zn^2+^. Yan et al. [19] also suggested that the H_2_O‐solvated Zn^2+^ had a largely reduced effective charge and thus weaker electrostatic interactions with the V_2_O_5_ framework, thereby promoting its diffusion. A facile in situ approach was developed by Zhao et al. [40] to simultaneously realize multivalence introduction, increased interlayer water content, and expanded interlayer space in hydrated V_2_O_5_ (from 11.6 to 14.0 Å); the structural water in V_2_O_5_ facilitates ion transport by reducing migration barriers to the diffusion of Zn^2+^. These results highlight the critical role of controlled dehydration in tuning interlayer structure and electrochemical performance [41]. Electrochemical characterization demonstrated that the as‐prepared samples without heat treatment showed rather poor cycling stability (only 56 mAh g^−1^ after 600 cycles at 0.5 A g^−1^). After being heat treated, particularly at 150 °C, the corresponding cell exhibited the best cycling stability (179 mAh g^−1^ after 600 cycles at 0.5 A g^−1^). When the heat treatment temperature was below 300 °C, VOH retained its hydrated phase, with the interlayer water content gradually decreasing as the temperature increased. Above 300 °C, VOH begins to depart from the hydrated phase transforming back to the orthorhombic phase (Figure 2), accompanied by a sharp reduction in the interlayer spacing. An optimum water content is a key parameter that enhances the electrochemical performance of VOH, since excess structural water can lead to weak interlayer forces and structural collapse of the active materials, thereby resulting in poor Zn^2+^ ion storage behavior.

The interlayer distance of M_*x*_V_2_O_5_·nH_2_O depends on the amount of interlayer water and the type of preinserted metal cation (M). Higher water content, larger hydrated ion radius, and greater electronegativity of M result in an expanded interlayer spacing. M_*x*_V_2_O_5_·nH_2_O with optimal structural water content exhibits superior electrochemical performance for aqueous ZIBs [42]. It is the synergistic effect between the intercalated metal‐ion and water molecules that boosts electrical conductivity, accelerates Zn^2+^ diffusion, and enhances structural stability during cycling [43, 44, 45]. In M_*x*_V_2_O_5_·nH_2_O, intercalated metal ions and/or structural water acted as pillars [46, 47, 48, 49, 50]. However, the crystal structure may show different structural evolutions or phase transitions depending upon the type(s) and amounts of intercalated metal ions.

## 2 Single Metal‐Ion Intercalation

**FIGURE 3:**
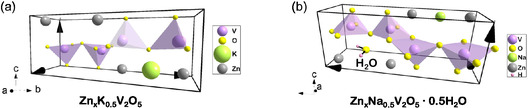
Crystal structures of V_2_O_5_ and VOH highlighting interlayer metal atoms and H_2_O. (a) Zn_*x*_K_0.5_V_2_O_5_ and (b) Zn_*x*_Na_0.5_V_2_O_5_·H_2_O. Original to this study.

Vanadium pentoxide possesses a typical layered structure (Figure 2) that enables metal‐ion storage; however, it suffers from poor structural stability and smaller interlayer or tunnel spacing (∼4.4–4.8 Å). The relatively weak van der Waals forces between the V_2_O_5_ layers can be easily broken (Figure 3a), making them more prone to degradation during the intercalation/deintercalation of Zn^2+^ ions. The introduction of monovalent, divalent, or trivalent metal ions, similar to structural water molecules, acts as interlayer pillars, which provides an effective method to enhance overall structural stability. In general, single metal ion intercalation in VOH modifies the interlayer environment of layered materials by expanding the interlayer spacing, altering the electronic and crystal structures, and, in some cases, serving as dopants. Consequently, single metal‐ion intercalation enhances the electrochemical properties, leading to enhanced capacity, energy and power density, and cycle stability.

### 2.1 Monovalent Metal‐Ion Intercalation

Monovalent metal ions exhibit weaker electrostatic interactions with the host lattice. Thus, the partial replacement of Zn^2+^ ions with monovalent guest metal ions could enhance the overall performance of aqueous ZIBs. The K^+^ intercalated V_2_O_5_ nanorods prepared by Islam et al. [51] exhibited significant discharge capacities of 439 mAh g^−1^ at 0.05 A g^−1^. Furthermore, it recovered 96% of the capacity after 1500 cycles at 8 A g^−1^. The K_0.5_V_2_O_5_ electrode prepared by Su et al. [52] achieved high reversible capacities of 241 and 115 mAh g^−1^ at 1 and 5 A g^−1^.

Intercalation of monovalent metal ions generally has an increased interlayer space compared to VOH. This increase in interlayer spacing enhances the material's conductivity and ion transport by increasing interlayer spacing and reducing ion–lattice interactions, thereby improving its experimental specific capacity. For example, Yang et al. [53] demonstrated that intercalating Li^+^ ions into VOH increased interlayer spacing and accelerated Zn^2+^ ion diffusion. Li et al. [54] prepared prepotassiated hydrated V_2_O_5_ in which the K^+^ acted as pillars to stabilize the lamellar structure, and crystal water facilitates ion transport and improves the diffusion efficiency of Zn^2+^ ions. Using a simple, effective hydrothermal method, Chen et al. [55] developed a bi‐intercalated structure of K^+^ and H_2_O in V_2_O_5_ with a large interlayer spacing (10.2 Å), facilitating efficient Zn^2+^ insertion and extraction. As the dosage of hydrogen peroxide increased, the water content also increased significantly, indicating that hydrogen peroxide effectively promoted the transformation of vanadium oxide into its hydrated phase. The modification of V_2_O_5_ with K^+^ not only increased the interlayer spacing but also improved the conductivity of vanadium oxide, which further enhanced the Zn^2+^ diffusion kinetics [56] (Figure 3b).

But in some cases, intercalation of monovalent metal ions results in a decrease in the interlayer spacing compared to VOH, mainly due to the strong interaction between monovalent metal ions and lattice oxygen in interlayers. Jia et al. [57] synthesized Na_0.27_V_2_O_5_⋅0.7H_2_O by preintercalating Na^+^ ions (Figure 3), replacing approximately 0.3 interlayer water molecules per V_2_O_5_ unit. This substitution reduced the interlayer spacing from 12.0 (in VOH) to 11.0 Å, yet enabled faster Zn^2+^ diffusion, delivering a high capacity of 420 mAh g^−1^ at 0.05 A g^−1^, 88% energy efficiency, and excellent cycling stability (88% retention after 2000 cycles at 4 A g^−1^) compared to VOH. The enhanced performance of Na_0.27_V_2_O_5_⋅0.7H_2_O stems from reduced structural water content, increased V^4+^ concentration, improved electron hopping between V^4+^ and V^5+^, and weaker Zn^2+^–host interactions, allowing smoother and more reversible ion transport. This demonstrates that reduced solvation and optimized electronic structures govern Zn^2+^ transport.

Optimal performance is achieved for intermediate ionic radii (e.g., K^+^), which balance structural expansion and ion mobility. KV_12_O_30‐y_·nH_2_O, reported by Tian et al. [58], led to a contracted interlayer spacing compared to VOH. Qi et al. [59] developed a Cs^+^ ion intercalated V_2_O_5_ cathode, where the strong Cs–O interaction reduced the interlayer spacing to 10.8 Å, compared to 12.1 Å in VOH, thus stabilizing the layered structure and significantly enhancing cycle life (89% capacity retention over 10,000 cycles at 20 A g^−1^). This Cs^+^ intercalated material exhibited superior specific capacities of 404.9 mAh g^−1^ at 0.1 A g^−1^ and 189.9 mAh g^−1^ at 20 A g^−1^, outperforming the pristine VOH electrode. The interlayer spacing of vanadium oxide can be correlated with the interlayer water content along with the electronegativity, ionic radius, and concentration of the preintercalated metal‐ion.

Among the monovalent metal ion intercalated V_2_O_5_ cathodes summarized in Table 1, K‐ and Cs‐modified systems demonstrate the most balanced electrochemical characteristics when capacity, rate performance, and long‐term durability are considered collectively. In particular, K_0.5_V_2_O_5_.nH_2_O exhibits very high reversible capacity together with sustained cycling behavior, indicating efficient Zn^2+^ storage within a structurally stabilized layered framework [56]. Alkali metals have an outer ns1 configuration, and their ionic radius increases down the group. In layered vanadium oxide cathodes, Li^+^ is too small and strongly polarizing, which can cause lattice distortion and slow diffusion, while Na^+^ offers only moderate structural expansion. K^+^ provides an optimal ionic size that expands the interlayer spacing without destabilizing the framework, thereby facilitating Zn^2+^ diffusion and improving electronic transport. Larger ions such as Rb^+^ and Cs^+^ may hinder ion mobility due to their size, and Fr^+^ is radioactive, making K^+^ the most effective monovalent metal ion for enhancing electrochemical performance. In group 1, Rb^+^ and Fr^+^ have not been explored for monovalent metal ion intercalation because Fr^+^ is highly radioactive and unstable, while Rb^+^ has a large ionic size that can distort the layered structure and slow ion diffusion, in addition to being costly and impractical for battery applications. Apart from alkali metal ions, a few other monovalent‐ions like H^+^, NH^4+^, organic monovalent cations, etc., can be intercalated into layered V_2_O_5_ systems under specific conditions. H^+^ cointercalation often occurs together with Zn^2+^ in ZIBs and can improve kinetics (Figure 2b, left), whereas NH^4+^ is used as a structure directing or pillaring ion. Organic monovalent cations act as spacers but usually reduce energy density due to their large size.

**TABLE 1:** Electrochemical performance and interlayer spacing of monovalent metal‐ion intercalated V_2_O_5_ cathode materials.

Material	Capacity, mAh g^−1^	Capacity retention	Electrolyte	Interlayer spacing, Å	Voltage window, V	Synthesis method	**Reference,** **year**
LVO‐250	470.00 at 0.5 A g^−1^	232 mAh g^−1^ after 500 cycles at 5.0 A g^−1^	2 M ZnSO_4_	13.77	0.4–1.4	Hydrothermal	[53], 2018
*δ*‐Na_0.27_V_2_O_5_ ⋅0.7H_2_O	420.00 at 0.05 A g^−1^	88.00% after 2000 cycles at 4 A g^−1^	3 M Zn(CF_3_SO_3_)_2_	11.00	0.2–1.6	Hydrothermal	[57], 2022
KV_12_O_30‐y_⋅ nH_2_O	436.00 at 0.05 A g^−1^	92.00% after 3000 cycles at 5 A g^−1^	3 M Zn(CF_3_SO_3_)_2_	9.90	0.2–1.6	Hydrothermal	[58], 2020
KVO	439.00 at 0.05 A g^−1^	96.00% after 1500 cycles at 8 A g^−1^	1 M ZnSO_4_	9.40	0.4–1.4	Hydrothermal	[51], 2019
K_0.52_V_2_O_5_ ⋅0.29H_2_O	270 at 0.1 A g^−1^	100.00% after 1500 cycles at 2 A g^−1^	PVDF‐Zn(ClO_4_)_2_based polymer membrane (quasisolid electrolyte)	10.4	0.4–1.4	Microwave‐assisted hydrothermal	[54], 2022
KVOH	415.30 at 0.1 A g^−1^	95.90% after 800 cycles at 0.1 A g^−1^	3 M Zn(OTf)_2_	10.20	0.2–1.6	Hydrothermal	[55], 2024
K_0.5_V_2_O_5_	486.00 at 0.1 A g^−1^	251 mAh g^−1^ after 1000 cycles at 5 A g^−1^	3 M Zn(CF_3_SO_3_)_2_	9.48	0.2–1.6	Hydrothermal	[52], 2021
K_0.5_V_2_O_5_⋅ nH_2_O	372.70 at 1 A g^−1^	66.90% after 9000 cycles at 10 A g^−1^	0.005 M KCl	10.60	0.2–1.6	Hydrothermal	[56], 2023
CsVO	404.90 at 0.1 A g^−1^	89.00% after 10000 cycles at 20 A g^−1^	3 M Zn(CF_3_SO_3_)_2_	10.80	0.2–1.6	Hydrothermal	[59], 2022

### 2.2 Divalent Metal‐Ion Intercalation

**FIGURE 4:**
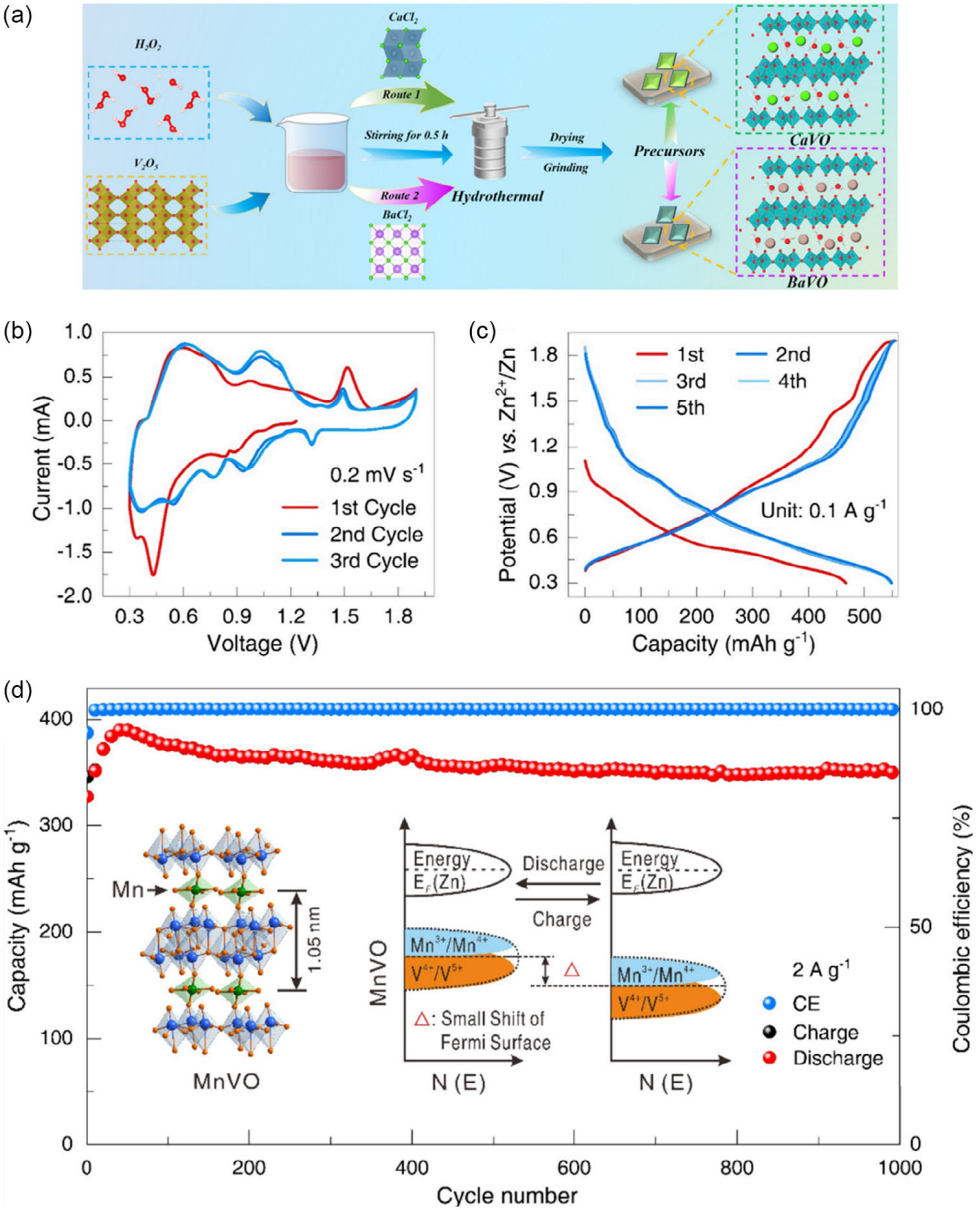
Effects of interlayer spacing of divalent metal‐ion intercalated V_2_O_5_ cathode materials. (a) Schematic illustration of synthesis of Ca^2+^ and Ba^2+^ into vanadium oxides, reproduced with permission [61], Copyright 2024, Elsevier. (b) CV plots were obtained for the initial three cycles of the Mn_0.28_V_2_O_5_·0.97H_2_O cathode using a scanning rate of 0.2 mV s^−1^, (c) GCD profiles at a current density of 0.1 A g^−1^ for the Mn_0.28_V_2_O_5_·0.97H_2_O cathode, (d) the cycling properties with crystal structure and energy versus density of states (DOS) schematics of Mn_0.28_V_2_O_5_·0.97H_2_O at current density of 2 A g^−1^, reproduced with permission [47], Copyright 2024, ACS.

Unlike monovalent metal ions, which have lower charge density, divalent metal ions have stronger Coulombic interactions, leading to enhanced structural stabilization but higher diffusion barriers, resulting in a more stable structure for vanadium oxides. Although divalent metal ions exhibit similar functional principles to monovalent metal ions, the large radius of these ions is more beneficial to Zn^2+^ intercalation. The stable chemical bonding between the Ca and O atoms is larger than that of Zn–O, which helps to attain reversible Zn^2+^ insertion and fast ion transport in Ca_0.25_V_2_O_5_·nH_2_O by Xia et al. [60] and Ca_0.04_V_2_O_5_⋅1.74H_2_O by Du et al [43]. In hydrated vanadium oxides with preintercalated Mn^2+^ ions, Mn^2+^ and H_2_O together act as pillars that support the basic structure between the vanadium oxygen layers [46, 47]. They have demonstrated a high reversible specific capacity and a long‐term cycling stability. Mn_0.19_V_2_O_5_·nH_2_O achieved a reversible capacity of 394.54 mAh g^−1^ at 0.2 A g^−1^, whereas Mn_0.28_V_2_O_5_·0.97H_2_O of 549 mAh g^−1^ at 0.1 A g^−1^ (Figure 4b–d).

The crystal structure and morphology of Zn_0.1_V_2_O_5_⋅0.7H_2_O were well maintained even after soaking in ZnSO_4_ aqueous electrolyte for 30 days, indicating the high compatibility with aqueous electrolytes [62]. Ca containing bilayered vanadium oxide as a cathode material for ZIBs showed an outstanding performance in wet organic electrolytes [63]. Geng et al. [44] group's Mn_0.15_V_2_O_5_·0.72H_2_O composites with a narrowed direct bandgap achieved a specific capacity of 100 mAh g^−1^ at 2.0 A g^−1^ after 3000 cycles, even at −20 °C. A nondrying, antifreezing, tough, flexible battery with high energy density was constructed by Ma et al [48].

Generally, a hydrothermal approach gives metal‐ion intercalated hydrated vanadium oxides, but Kundu et al. [49] utilized a microwave‐assisted approach for the synthesis of Zn_0.25_V_2_O_5_⋅nH_2_O nanobelts. Unlike typical hydrothermal methods, microwave heating enables rapid and localized dipolar heating of water, causing uniform and fast heating of the reaction medium that triggers the intercalation and exfoliation of V_2_O_5_ into Zn_*x*_V_2_O_5_⋅nH_2_O nanobelts. Ren et al. [64] prepared Zn‐ions and water intercalated V_2_O_5_ nanowires by one‐step stirring. Similarly, Pang et al. [65] introduced metal‐ion inserted vanadium oxide (MVO, M = Zn, K, Na, and Ca) nanoribbons by one‐step stirring, where this synthesis strategy can be easily scaled up.

Zhou et al. [61] compared the intercalation of similar divalent metal ions (Ca^2+^ or Ba^2+^) in vanadium oxides (Figure 4) and found that Ca^2+^ intercalated V_2_O_5_ outperformed both Ba^2+^ intercalated V_2_O_5_ and pristine V_2_O_5_ in electrochemical performance. This work has also provided insight into zinc storage performance determined by multicomponent synergy. Liu et al. [66] examined the catalytic roles of Cu(II) and Mg(II) metal ions in electrochemical performance and found that Cu(II) significantly enhanced zinc‐ion intercalation kinetics, even though their ionic radius is very close. Thus, the radii of the metal ions alone are unlikely to be the sole determining factor in the change in interlayer spacing, with electronic structure and bonding playing a dominant role.

Yang et al. [38] further explored the preintercalation of various transition metal ions (Fe^2+^, Co^2+^, Ni^2+^, Mn^2+^, Zn^2+^, and Cu^2+^) into V_2_O_5_ and identified Cu_0.1_V_2_O_5_⋅0.08H_2_O (CuVO) as the most effective. Although transition metal preintercalation during the hydrothermal process did not maximize interlayer spacing (CuVO: 11.45 Å versus VO: 13.83 Å), the enhanced thermal stability of CuVO allowed the lattice structure to remain stable postannealing (CuVO at 300 °C: 11.45 Å versus V_2_O_5_ at 300 °C: 10.32 Å).

The amount of crystal water in the interlayer space of vanadium oxide also has an important effect on the electrochemical performance. By adjusting the Zn‐to‐V ratio, Gu et al. [42] optimized the crystal water content of Zn_*x*_V_2_O_5_⋅nH_2_O, resulting in Zn_0.146_V_2_O_5_⋅0.579H_2_O, which showed superior electrochemical performance.

By adjusting the ratio of the doped metal ion, the structural parameters and interlayer spacing of the material can be controlled. By increasing the Fe content, the sample structure transformed from nanowires to nanobelts [45]. However, too many preintercalated ions can occupy the active Zn storage sites, thereby influencing the battery performance. Increased Mn doping not only hinders ion intercalation/extraction but also leads to structural collapse during cycling [67].

Divalent metal ion intercalation in V_2_O_5_ introduces a stronger electrostatic interaction with the host lattice compared to monovalent systems, resulting in enhanced stabilization but adds complexity to diffusion behavior due to higher charge density. Among the reported materials in Table 2, Cu_0.1_V_2_O_5_⋅0.08H_2_O showed excellent specific capacity due to enhanced electronic conductivity and the pillaring effect of Cu^2+^, along with the strong Cu—O bond, which stabilizes the framework [38]. Although Cu^2+^ preintercalation during the hydrothermal process did not maximize interlayer spacing, the enhanced thermal stability of Cu_0.1_V_2_O_5_⋅0.08H_2_O allowed the lattice structure to remain stable postannealing.

**TABLE 2:** Electrochemical performance and interlayer spacing of divalent metal‐ion intercalated V_2_O_5_ cathode materials.

Material	Capacity, mAh g^−1^	Capacity retention	Electrolyte	Interlayer spacing, Å	Voltage window, V	Synthesis method		Reference, year	
Ca_0.26_V_2_O_5_⋅H_2_O	268.76 at 0.05 A g^−1^	83.00% after 120 cycles at 0.5 A g^−1^	0.5 M Zn(ClO_4_)_2_ solution in a wet organic solvent comprising Facetonitrile	11.02	0.5–1.3	Hydrothermal		[63], 2023	
Ca_0.25_V_2_O_5_·nH_2_O	340.00 at a rate of 0.2C	96.00% after 3000 cycles at 80C	1 M ZnSO_4_	—	0.6–1.6	Hydrothermal		[60], 2018	
Ca_0.04_V_2_O_5_⋅1.74H_2_O	400.00 at 0.05 A g^−1^	100.00% after 3000 cycles at 10 A g^−1^	3 M Zn(CF_3_SO_3_)_2_	12.48	0.2–1.6	Hydrothermal		[43], 2020	
Ca_0.15_V_2_O_5_⋅2.2H_2_O	489.80 at 0.1 A g^−1^	201.2 mAh g^−1^ after 1000 cycles at 3 A g^−1^	3 M Zn(CF_3_SO_3_)_2_	10.58	0.2–1.6	Hydrothermal		[61], 2024	
Mn_0.2_V_8_O_20_⋅ 1.12H_2_O	306.40 at 0.1 A g^−1^	86.40% after 1000 cycles at 2 A g^−1^	2 M Zn(CF_3_SO_3_)_2_	10.30	0.2–1.6	Hydrothermal		[67], 2023	
Mn_0.15_V_2_O_5_· 0.72H_2_O	367.00 at 0.1 A g^−1^	153 mAh g^−1^ after 8000 cycles at 10 A g^−1^	1 M Zn(ClO_4_)_2_ in propylene carbonate (PC)	13.26	0.2–1.7	Hydrothermal		[44], 2020	
Mn_0.19_V_2_O_5_·nH_2_O	394.54 at 0.2 A g^−1^	87.00% after 960 cycles at 4 A g^−1^	3 M Zn(CF_3_SO_3_)_2_	12.70	0.2–1.6	Hydrothermal		[46], 2023	
Mn_0.28_V_2_O_5_· 0.97H_2_O	549.00 at 0.1 A g^−1^	91.00% after 100 cycles 0.2 A g^−1^	3 M Zn(CF_3_SO_3_)_2_	10.50	0.3–1.9	Hydrothermal		[47], 2024	
Fe_0.13_V_2_O_5_⋅nH_2_O	427.30 at 0.3 A g^−1^	102.00% after 1000 cycles	2 M Zn (CF_3_SO_3_)_2_	11.00	0.1–1.6	Hydrothermal		[45], 2024	
Co_0.247_V_2_O_5_⋅ 0·944H_2_O	432.00 at 0.1 A g^−1^	90.26% after 7500 cycles at 4 A g^−1^	20 M LiTFSI along 1 M Zn(TFSI)_2_	10.70	0.5–2.3	Hydrothermal		[48], 2019	
CuVOH	379.00 at 0.5 A g^−1^	93.00% after 1000 cycles at 4 A g^−1^	3 M Zn(CF_3_SO_3_)_2_	11.20	0.2–1.6	Hydrothermal		[66], 2020	
Cu_0.1_V_2_O_5_⋅ 0.08H_2_O	359.00 at 1 A g^−1^	88.00% after 10 000 cycles at 10 A g^−1^	2 M ZnSO_4_	11.45	0.3–1.4	Hydrothermal and subsequent annealing		[38], 2019	
ZnVO⋅1.2H_2_O	307.10 at 0.1 A g^−1^	100.00% after 3000 cycles at 4 A g^−1^	3 M Zn(CF_3_SO_3_)_2_	—	0.2–1.6	Stirring		[64], 2023	
ZnVO	414.80 at 0.1 A g^−1^	84.40% after 1500 cycles at 2 A g^−1^	3 M Zn(CF_3_SO_3_)_2_	—	0.3–1.3	Stirring		[65], 2022	
Zn_0.146_V_2_O_5_⋅ 0.579H_2_O	416.00 at 0.1 A g^−1^	105.00% after 10 000 cycles at 10 A g^−1^	3 M Zn(CF_3_SO_3_)_2_	13.70	0.3–1.4	Hydrothermal and subsequent freeze‐drying		[42], 2022	
Zn_0.1_V_2_O_5_⋅ 0.7H_2_O	463.00 at 0.2 A g^−1^	88.00% after 20 000 cycles at 10 A g^−1^	2 M ZnSO_4_	10.40	0.3–1.7	Hydrothermal		[62], 2021	
Zn_0.25_V_2_O_5_⋅nH_2_O	220.00 at 4.5 A g^−1^	80.00% after 1000 cycles at 2.4 A g^−1^	1 M ZnSO_4_	—	0.5–1.4	Microwave‐assisted hydrothermal		[49], 2016	

These results highlight a tradeoff between structural stability and ion mobility. The other divalent metal ions such as Cd^2+^, Hg^2+^, Pb^2+^, Be^2+^, etc., are considered toxic and are hence rarely explored in this context.

### 2.3 Trivalent Metal‐Ion Intercalation

Preintercalation with higher valence state ions has also been shown to act as structural pillars, thereby enhancing stability. The morphology of the electrode material has an influence on the electrochemical performance of aqueous ZIBs, for example, a coral‐like morphology consisting of thin nanosheets through a one‐step hydrothermal process of Cr‐VOH resulted in the fast Zn^2+^ migration [68]. Dosage of H_2_O_2_ during synthesis governs the micromorphology of Al_0.15_V_2_O_5_⋅1.01H_2_O [50]. As the H_2_O_2_ content increased, the pore size gradually enlarged, but exceeding 2 mL led to nanoparticle formation that blocked the pores and caused the disappearance of the tremella‐like morphology of Al_0.15_V_2_O_5_⋅1.01H_2_O. Similarly, the uniform urchin‐like morphology of Al‐doped V_10_O_24_⋅12H_2_O was in favor of a better electrochemical performance [69].

Hu et al. [70] synthesized yttrium‐ion intercalated VOH (Y–VOH) with a honeycomb porous morphology by a simple sol–gel method. This morphology increases electrolyte‐material contact area, thereby enhancing the electrochemical reactive sites to improve the energy storage capacity. Moreover, the honeycomb‐like porous spherical structure provides a large surface area and facilitates faster ion transport. A facile hydrothermal method was employed to synthesize Ru^3+^ intercalated vanadium oxide (Ru_0.2_V_2_O_5_·0.41H_2_O) with excellent rate performance and capacity retention [71].

Table 3 summarizes the electrochemical performance and interlayer spacing of trivalent metal ion intercalated V_2_O_5_ cathode materials. Here, Y–VOH with a honeycomb‐like porous spherical structure provided a large surface area and facilitated faster ion transport, resulting in a high specific capacity [70]. Sc^3+^, In^3+^, and Bi^3+^ are other trivalent metal ions, which can be intercalated into vanadium oxide to improve the electrochemical properties of ZIBs. The small trivalent metal ion Sc^3+^, has high mobility and higher charge density which can contribute to better diffusion and stability, whereas the moderate size In^3+^ having stable chemistry may show easier insertion than rare‐earth metal ions. Even though Bi^3+^ is heavy, it may enhance the electronic conductivity.

**TABLE 3:** Electrochemical performance and interlayer spacing of trivalent metal‐ion intercalated V_2_O_5_ cathode materials.

Material	Capacity, mAh g^−1^	Capacity retention	Electrolyte	Interlayer spacing, Å	Voltage window, V	Synthesis method	Reference, year
Al_0.15_V_2_O_5_· 1.01H_2_O	510.50 at 0.05 A g^−1^	144 mAh g^−1^ after 10,000 cycles at 10 A g^−1^	3M Zn(CF_3_SO_3_)_2_	14.19	0.1–1.8	Hydrothermal	[50], 2021
Al‐doped V_10_O_24_· 12H_2_O	415.00 at 0.2 A g^−1^	98.00% after 3000 cycles	3M Zn(CF_3_SO_3_)_2_	13.90	0.3–1.6	Hydrothermal	[69], 2019
Cr‐VOH	337.40 at 0.05 A g^−1^	58.10% after 500 cycles at 4 A g^−1^	3M Zn(CF_3_SO_3_)_2_	10.80	0.2–1.6	Hydrothermal	[68], 2021
Y–VOH	343.40 at 0.5 A g^−1^	90.00% after 3000 cycles	3M ZnSO_4_ along with 0.1 M VO^2+^	13.60	0.3–1.6	Sol–gel	[70], 2024
Ru_0.2_V_2_O_5_·0.41H_2_O	405.80 at 0.2 A g^−1^	98.20% after 5000 cycles at 10 A g^−1^	2M Zn(CF_3_SO_3_)_2_		0.2–1.6	Hydrothermal	[71], 2024

Several studies have reported the incorporation of hydrated metal‐ion into vanadium oxide layers which involves the insertion of metal‐ion together with their bound hydration shells as a single entity. Introduction of hydrated Mg^2+^ ions into vanadium oxides to obtain porous Mg_0.34_V_2_O_5_·0.84H_2_O nanobelts led to a broad operating voltage window of 0.1–1.8 V versus Zn^2+^/Zn, highlighting the structural benefits of hydrated metal‐ion intercalation [72]. Tong et al. [73] synthesized V_2_O_5_ nanoplates intercalated with hydrated Li^+^ ions, resulting in a stable lamellar structure with enlarged spacing, reduced interlayer repulsion, and lower molecular weight. A Mn_0.5_V_2_O_5_·2.4H_2_O cathode was developed by the incorporation of hydrated Mn^2+^ ions, which modulated the electronic structure, lowered Zn^2+^ migration barriers, and stabilized the layered framework for prolonged cycling life [74]. Similarly, the intercalation of hydrated Na^+^ ions into V_2_O_5_ not only enhances structural stability, but also lowers the desolvation energy barrier and accelerates ion intercalation kinetics [75]. Hydrated metal‐ion intercalation primarily augments structural stability and reduces electrostatic repulsion, whereas metal‐ion and water cointercalation primarily alters the charge storage mechanism by introducing fast proton kinetics and synergistic dual‐carrier transport, resulting in higher capacity, superior rate capability, and enhanced cycling stability.

Single preintercalated metal ions with different charge states, molecular weights, and ionic radii influence the electronic structure, mechanical properties, and ultimately the electrochemical performance in distinct ways. It is noteworthy that the appropriate choice of monovalent, divalent, or trivalent metal ions can increase the number of active sites. However, there is still a lack of systematic studies to determine the optimal choice of metal ion, as each interacts differently with layered vanadium oxides. Gaining a clearer understanding of the electrochemical behavior of guest‐ions in various electrolytes would be highly valuable in addressing these unresolved questions.

### 2.4 Metal Ions Intercalation Along With Carbon Additives

**FIGURE 5:**
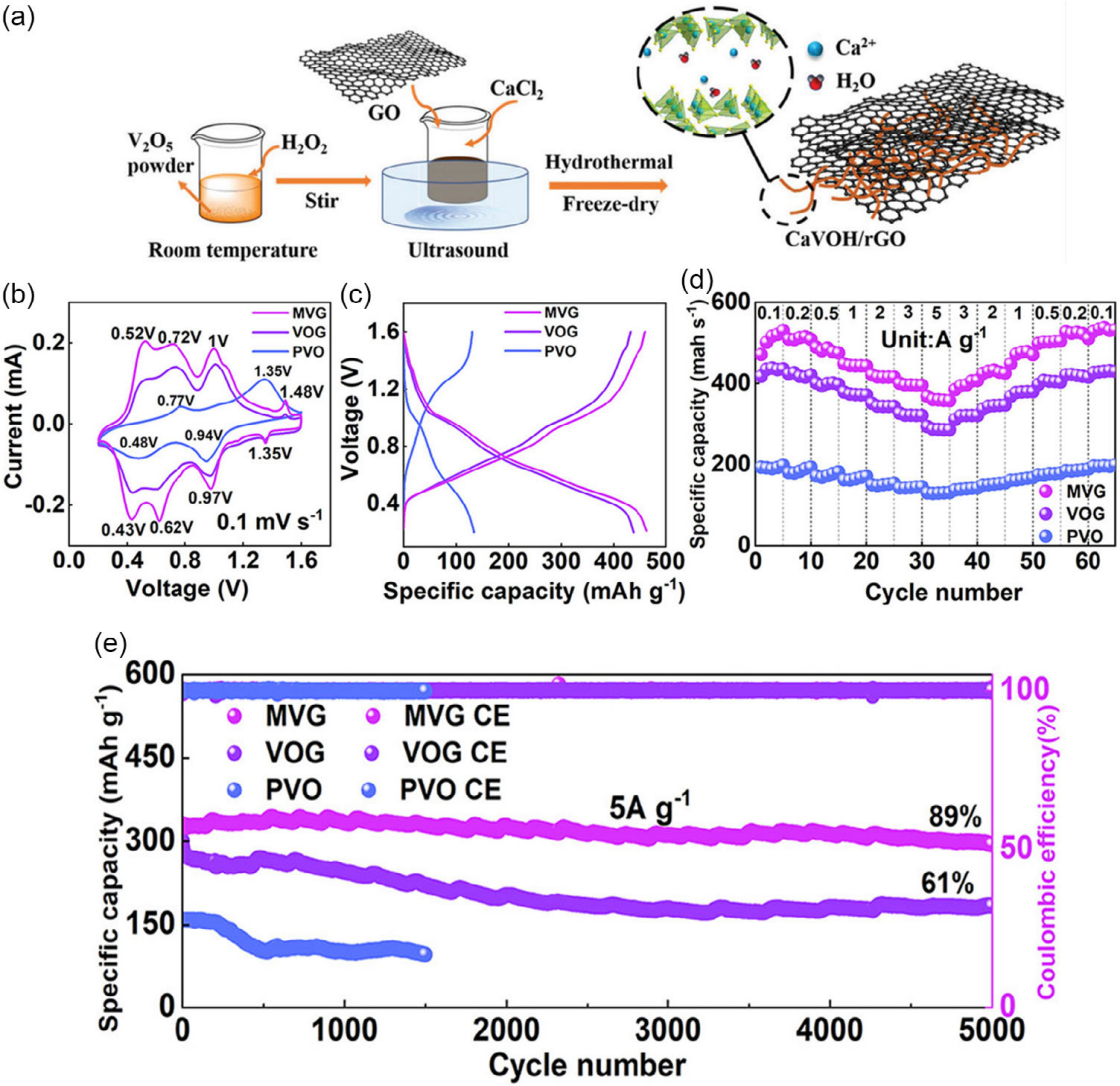
Calcium‐ions intercalation along with carbon additive. (a) Schematic illustration of the synthesis of Ca_0.22_V_2_O_5_⋅nH_2_O/rGO, reproduced with permission [76], Copyright 2021, RSC. (b) MVG, VOG, and PVO electrode CV curves at 0.1 mV s^−1^, (c) MVG, VOG, and PVO electrode GCD curves at 0.1 A g^−1^, (d) rate performance of MVG, VOG, and PVO electrodes at various current densities between 0.1 and 5.0 A g^−1^, and (e) cyclic performance of MVG, VOG, and PVO electrodes at 5 A g^−1^, reproduced with permission [77], Copyright 2024, ACS.

The inherently low conductivity of vanadium oxide results in substantial resistance polarization in aqueous ZIBs, while the stress from ion (de)intercalation can lead to structural collapse and degrade performance. Integrating vanadium oxide with conductive materials and intercalating metal ions have proven effective in enhancing electrode conductivity, facilitating ion transport, and reducing material dissolution. Graphene, a 2D carbon material, not only enhances the conductivity of electrodes but also stabilizes the original structure of active materials due to its fast electron transport capability and mechanical strength. For example, Ca^2+^‐intercalated VOH integrated with reduced graphene oxide (rGO)), synthesized via hydrothermal and freeze‐drying methods (Figure 5a), exhibited an enlarged interlayer spacing, improved structural stability, and enhanced conductivity [76]. Similar improvements were observed in Mn^2+^ inserted VOH nanobelts/rGO composites, where Mn^2+^ formed an electrostatic network with [VO_6_] polyhedra to facilitate Zn^2+^ diffusion, while rGO enhanced conductivity and energy density [78]. In Al_0.13_V_2_O_5_⋅nH_2_O/rGO, the expanded interlayer spacing promoted Zn^2+^ (de)intercalation, and the rGO coating improved conductivity and prevented vanadium dissolution [79]. When hybridized with Na_*x*_V_2_O_5_⋅nH_2_O, rGO significantly improved electrochemical performance compared to Na_2_V_6_O_16_⋅nH_2_O [80]. V_2_O_5_ nanoribbons wrapped with rGO and intercalated with Mg^2+^ ions demonstrated impressive electrochemical performance (Figure 5b–e), including a high specific capacity of 530 mAh g^−1^ at 0.1 A g^−1^ and outstanding capacity retention of 89% after 5000 cycles at 5 A g^−1^ [77].

A MnVO‐0.05–8/GN‐15 composite was synthesized, with Mn^2+^ increasing the interlayer spacing to 13.6 Å—up from 12.1 Å in pristine V_2_O_5_. After incorporating graphene, the spacing reduced to 11.6 Å, indicating that graphene partially penetrated and coated the MnVO structure. This integration resulted in faster Zn^2+^ diffusion and a high specific capacity of 507.5 mAh g^−1^ [81]. Carbon‐coated V_2_O_5_ nanoflowers with K^+^ preintercalation was hydrothermally prepared by reducing glucose as the carbon source, followed by high‐temperature annealing [82]. The annealing process improved the crystallinity of V_2_O_5_, particularly with increased glucose content during hydrothermal synthesis. With increasing glucose content, the lamellar structure on the edge of the nanoflower gradually shrinks, leading to a corresponding reduction in nanoflower size.

The integration of metal‐ion intercalated vanadium oxides with conductive carbon additives significantly enhances electrochemical performance by addressing the intrinsic limitations of vanadium oxide, particularly its low conductivity and structural instability during repeated Zn^2+^ insertion/extraction. Among these systems (Table 4), Mn‐based vanadium oxides coupled with graphene networks (MnVO‐0.05–8/GN‐15) demonstrated the most remarkable electrochemical behavior, as the redox activity of Mn^2+^ combined with the high electrical conductivity of graphene creates a synergistic framework that accelerates both ionic and electronic transport [81].

**TABLE 4:** Electrochemical performance and interlayer spacing of metal‐ion intercalated V_2_O_5_ cathode materials combined with carbon additives.

Material	Capacity, mAh g^−1^	Capacity retention	Electrolyte	Interlayer spacing, Å	Voltage window, V	Synthesis method	Reference, year
Ca_0.22_V_2_O_5_⋅nH_2_O/rGO	409.00 at 0.05 A g^−1^	90.00% after 2000 cycles at 4.0 A g^−1^	3 M Zn(CF_3_SO_3_)_2_	13.30	0.2–1.5	Hydrothermal and subsequent freeze‐drying	[76], 2021
MnVOH/rGO	380.00 at 0.1 A g^−1^	80.00% after 100 cycles	3 M Zn(CF_3_SO_3_)_2_	9.09	0.2–1.6	Hydrothermal	[78], 2021
KVO‐C3	389.20 at 0.1 A g^−1^	77.00% after 2000 cycles	2 M Zn(CF_3_SO_3_)_2_		0.0–1.6	Hydrothermal and subsequent annealing	[82], 2022
Al_0.13_V_2_O_5_⋅nH_2_O/rGO	405.00 at 0.1 A g^−1^		3 M Zn(CF_3_SO_3_)_2_	13.30	0.2–1.5	Hydrothermal and subsequent freeze‐drying	[79], 2022
rGO/Na_0.48_V_2_O_5_⋅0·36H_2_O	433.50 at 0.1 A g^−1^	70.50% over 1000 cycles	3 M Zn(CF_3_SO_3_)_2_	10.60	0.2–1.6	Hydrothermal and subsequent freeze‐drying	[80], 2019
MVG	530.00 at 0.1 A g^−1^	89.00% after 5000 cycles at 5 A g^−1^	3 M Zn(CF_3_SO_3_)_2_		0.2–1.6	Hydrothermal and subsequent freeze‐drying	[77], 2024
MnVO‐0.05–8/GN‐15	530.10 at 0.1 A g^−1^	85.70% after 2000 cycles at 3 A g^−1^	3 M Zn(CF_3_SO_3_)_2_	11.60	0.4–1.6	Hydrothermal	[81], 2024

An increase in active material load leads to agglomeration and shedding. However, directly growing active materials on conductive substrates with large surface areas helps prevent agglomeration and shedding, allowing higher mass loading per unit area. Advanced nanostructures such as nanowires, plates, columns, flower arrays increase active surface area, and expose more reactive sites. This enhances mass transfer, reaction kinetics and also ensures greater material utilization. This approach significantly boosts capacity, rate performance, and cycling stability. For example, calcium‐ion intercalated vanadium oxide grown on carbon cloth (CaVOH@CC) showed a high mass load (∼7 mg cm^−2^), specific capacity of 228.5 mAh g^−1^ at 1 A g^−1^, and 70% capacity retention after 800 cycles [83]. Sodium‐intercalated vanadium oxide nanostructures anchored on carbon cloth (Na30V@CC) also exhibited superior capacity, rate capability, and stability [84]. Thus carbon assisted, metal‐ion intercalated vanadium oxides represent a highly promising electrode architecture for next‐generation aqueous ZIBs, particularly for applications requiring high energy density, rapid charge discharge capability, and long operational lifetimes.

### 2.5 Metal Ions Intercalation Along With Defect Engineering

**FIGURE 6:**
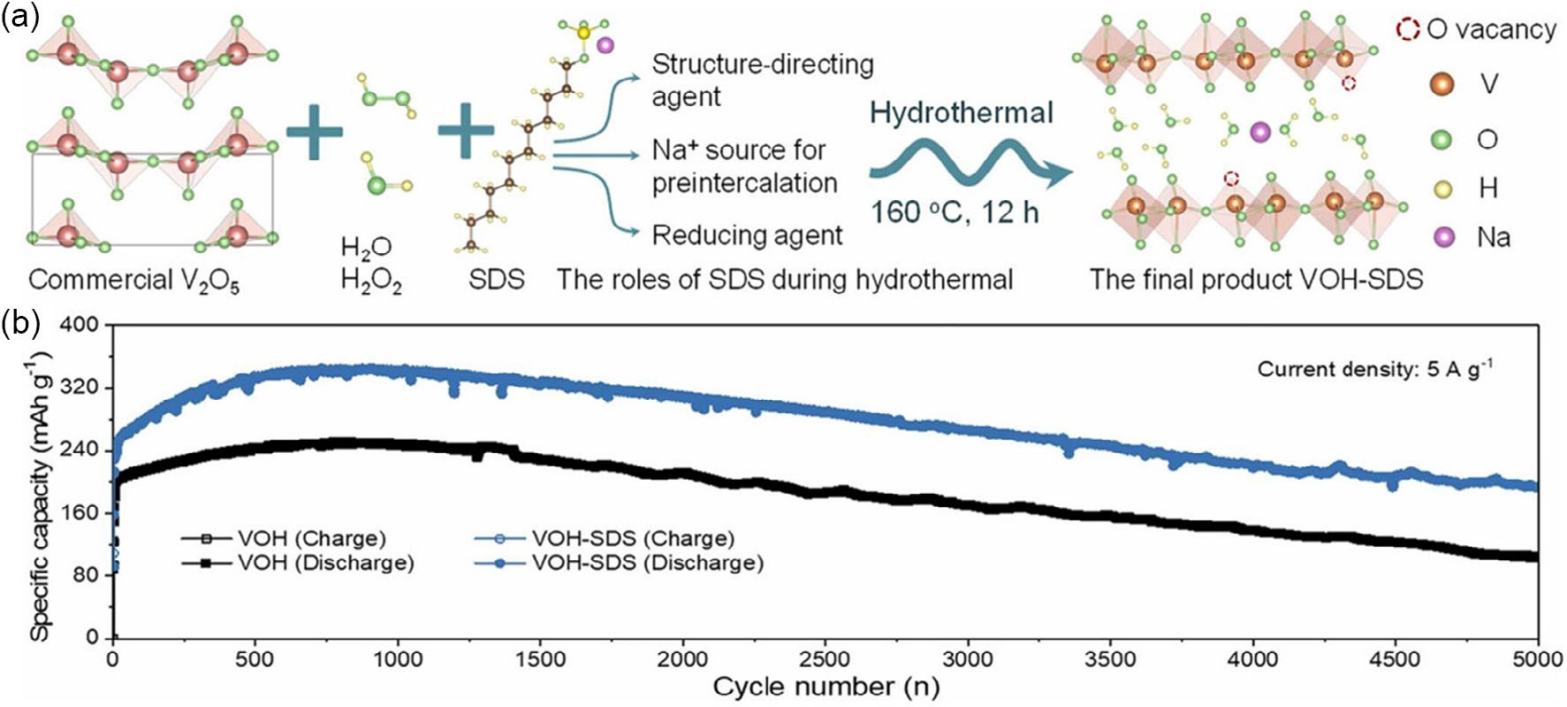
Effect of H_2_O, and H_2_O_2_ intercalation along with SDS additive source of Na^+^. (a) Schematic illustration of the preparation process of VOH‐SDS sample and (b) long‐term cycling performance curves of VOH‐SDS and VOH samples, reproduced with permission [86], Copyright 2024, Elsevier.

Introducing appropriate defects into the cathode materials is an effective strategy to enhance the electrical conductivity and electrochemical activity by tuning the distribution of localized electrons and the number of active sites. In vanadium oxides, similar improvements can be achieved by inducing atomic‐scale defects, such as oxygen vacancies (anion defects) and vanadium vacancies (cation defects). The large formation energy of metal‐cation vacancies renders their introduction challenging, and precise control of these vacancies remains difficult [85]. Hence, metal‐cation defective V‐based cathodes for ZIBs are much rarer than anion defects. However, cationic or anionic defects alone have a limited impact on improving electrochemical performance. Therefore, integrating interlayer and defect engineering presents a promising approach to develop high‐capacity, stable vanadium‐based layered oxide cathodes for ZIBs. Li et al. [86] synthesized VOH nanobelts using a simple hydrothermal method assisted by sodium dodecyl sulfate (SDS), as shown in Figure 6a. The SDS not only guided the formation of the nanobelt morphology but also promoted the generation of abundant oxygen vacancies and supplied Na^+^ ions for preintercalation. As a result of these combined advantages, the nanobelts demonstrated outstanding long‐term cycling stability, delivering a capacity of 193 mAh g^−1^ after 5000 cycles at a current density of 5 A g^−1^ (Figure 6b). Mn_1.4_V_10_O_24_·12H_2_O with oxygen defect prepared by a spontaneous chemical synthesis, delivered a high specific capacity of 456 mAh g^−1^ at 0.2 A g^−1^ [87].

An optimal amount of metal‐ion intercalation maximizes electrochemical performance by synergistically stabilizing the layered structure, increasing oxygen/vanadium vacancy concentration, and accelerating Zn^2+^ diffusion. For example, with a moderate amount of Cr^3+^ incorporation, Cr_0.27_V_2_O_4.8_⋅0.56H_2_O is conductive to Zn^2+^ diffusion while maintaining structural stability [88]. In K_0.39_V_2_O_5_·0.52H_2_O cathode, with optimized amounts of K^+^ doped and hydrated vanadium oxide, pre‐embedding of K^+^ ions expanded the interlayer spacing and stabilized the crystal structure, while the presence of oxygen vacancies created additional active sites for Zn^2+^ storage [89]. Gao et al. [90] developed calcium‐intercalated hydrated vanadium oxide nanobelts featuring cationic vanadium vacancies.

Table 5 presents the electrochemical performance and interlayer spacing of metal‐ion intercalated V_2_O_5_ cathode materials incorporating defect engineering. Cr_0.27_V_2_O_4.8_⋅0.56H_2_O, containing a moderate amount of Cr^3+^ incorporation, has better specific capacity due to the synergistic effects of structural stabilization, defect engineering (oxygen/vanadium vacancies), and accelerated Zn^2+^ diffusion [88].

**TABLE 5:** Electrochemical performance and interlayer spacing of metal‐ion intercalated V_2_O_5_ cathode materials incorporating defect engineering strategies.

Material	Capacity, mAh g^−1^	Capacity retention	Electrolyte	Interlayer spacing, Å	Voltage window, V	Synthesis method	Reference, year
K_0.39_V_2_O_5_· 0.52H_2_O	552.00 at 0.1 A g^−1^	90.00% after 11 000 cycles at 10 A g^−1^	3 M Zn(CF_3_SO_3_)_2_	11.00	0.3–1.6	Hydrothermal	[89], 2025
VOH‐SDS	363.00 at 0.2 A g^−1^	193 mAh g^−1^ after 5000 cycles at 5 A g^−1^	3 M ZnSO_4_	12.40	0.0–1.6	Hydrothermal and subsequent freeze‐drying	[86], 2024
Mn_1.4_V_10_O_24_·12H_2_O	456.00 at 0.2 A g^−1^	80.00% after 5000 cycles at 10 A g^−1^	3 M Zn(CF_3_SO_3_)_2_	13.50	0.2–1.6	Spontaneous chemical reaction	[87], 2021
CaVO‐5	310.00 at 0.5 A g^−1^	91.70% after 3000 cycles at 10 A g^−1^	2 M ZnSO_4_	12.58	0.0–1.6	Hydrothermal	[90], 2022
Cr_0.27_V_2_O_4.8_⋅0·56H_2_O	405.00 at 0.5 A g^−1^	120.00% after 3500 cycles	3 M Zn(CF_3_SO_3_)_2_		0.2–1.4	Hydrothermal	[88], 2023

Some groups even integrated vanadium oxide with conductive materials along with intercalating metal ions and introducing defects, like glucose‐derived hydrothermal carbon (GHC) stabilized and transition metal‐ion Mn doped hydrated vanadium oxide composite material [91]. Oxygen vacancies generated by the surface‐anchored GHC and interlayer‐inserted Mn^2+^ enhanced the number of active sites, improved electronic conductivity, and facilitated ion diffusion. In this structure, GHC maintained external structural integrity, while Mn^2+^ served as a pillar to support the interlayer framework. As a result, the Mn‐VOH@GHC electrode delivered a high capacity of 530 mAh g^−1^ at 0.2 A g^−1^. Such synergistic design—integrating interlayer tuning, vacancy control, and conductive framework support—could potentially push the capacity while maintaining ultra‐long cycle life and high‐rate capability.

## 3 Cointercalation of Metal Ions and Other Additives

### 3.1 Cointercalation of Metal Ions and Organic Additives

**FIGURE 7:**
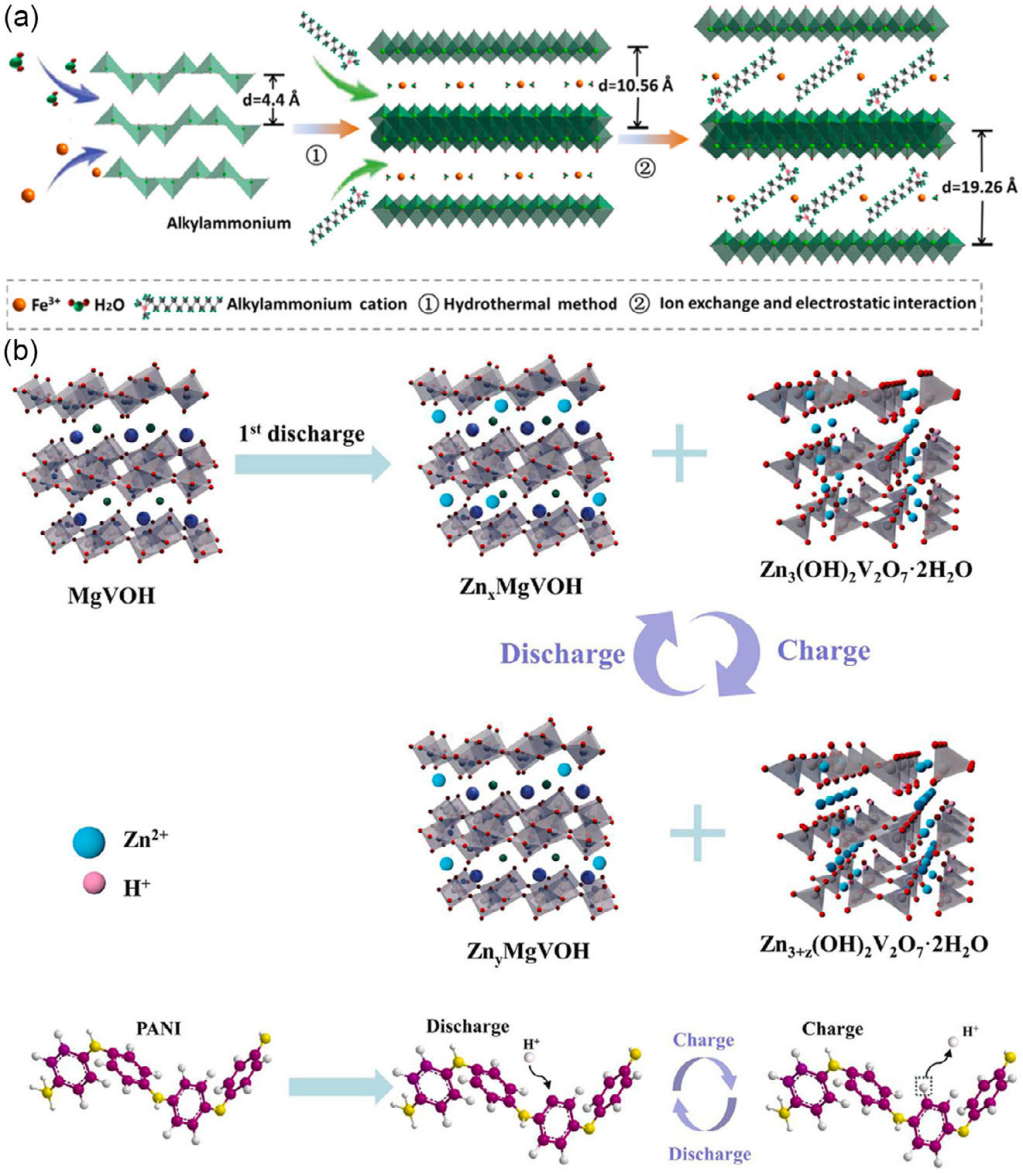
Effect of trivalent intercalation, hydration, and additives. (a) Schematic illustration of iron‐ion and alkylammonium cation cointercalated vanadium oxide (FeVO‐12) by a hydrothermal method, followed by ion‐exchange and electrostatic interaction, reproduced with permission [93], Copyright 2022, Elsevier. (b) Schematic illustration of the reaction mechanism of the MgVOH/PANI electrode, reproduced with permission [94], Copyright 2024, Elsevier.

Organic additives are intercalated into various layered materials to regulate the structure and performance of host materials. Organic additives have larger ionic radii/molecular dimensions than those of metal ions and can effectively manipulate the interlayer spacing of vanadium oxides by adjusting their molecular dimensions. This reduces electrostatic interactions with Zn^2+^ ions and the VO frameworks which directly enhances Zn^2+^ diffusion, and offers more active sites for charge accommodation. Indeed, the metal ions and organic cations seem to act concurrently to enhance capacity by partially reducing V^5+^ to V^4+^ and/or introducing oxygen vacancies to maintain electro neutrality, though they serve distinct roles when intercalated. A composite material with polyethylene glycol (PEG) and Ba^2+^ ions preintercalated into V_2_O_5_ layers was developed, achieving excellent electrochemical performance [92]. Cointercalation widened the interlayer spacing between adjacent VO layers to 10.7 Å, thereby enhancing Zn^2+^ transport kinetics. Theoretical studies confirmed the structure's thermodynamic stability, while PEG‐Ba^2+^ interactions increased the V^4+^/V^5+^ ratio, thereby improving structural stability and electronic conductivity. Tong et al. [93] successfully synthesized an iron‐ion and alkylammonium cation cointercalated vanadium oxide through a two‐step intercalation process, as illustrated in Figure 7a. The intercalated iron‐ions enhanced structural stability through electrostatic interactions, while the alkylammonium cations expanded the interlayer spacing and preserved surface hydrophobicity. Together, these effects contributed to a high specific capacitance and excellent cycling stability. Due to the synergistic effect of cointercalated Zn^2+^ ions and n‐butylamine, the cointercalated sample ZBVO exhibited exceptional electrochemical performance [95]. The Zn^2+^ ions formed strong Zn—O bonds that acted as structural pillars, while n‐butylamine preserved optimal interlayer spacing, enabling efficient and reversible Zn^2+^ diffusion. Vanadium oxide co‐preinserted with N(CH_3_)_4_^+^ and Zn^2+^ ions has been reported as a cathode for ultra‐stable aqueous ZIBs [96]. The presence of organic N(CH_3_)_4_^+^ ions reduce electrostatic interactions between Zn^2+^ and the host structure, thereby enhancing the reversibility of Zn^2+^ insertion and extraction and extending cycle life. Guo et al. [97] introduced inorganic Al^3+^ ions and amphoteric organic‐ions (betaine) into VOH to create an inorganic–organic cointercalated cathode. Both Al^3+^ and betaine contributed to expanding the interlayer spacing and stabilizing the layered structure. The quaternary ammonium groups in betaine formed strong interactions with lattice oxygen of V_2_O_5_, enhancing structural stability, while the polar carboxyl groups weakened Zn^2+^–V–O interactions, thereby facilitating improved Zn^2+^ diffusion.

The conductive polymer PANI significantly enhances rate capability by constructing electron‐ transport networks. Hu et al. [94] developed a MgVOH/PANI cathode by cointercalating Mg^2+^ ions and the conductive polymer PANI into hydrated vanadium oxide layers. Mg^2+^ improved conductivity and expanded interlayer spacing, while PANI mitigated structural strain during cycling and stabilized the layered framework. Additionally, PANI participated in redox reactions (Figure 7), enhancing Zn^2+^ diffusion and overall electrochemical performance. The cointercalation of K^+^ ions and PANI into VOH demonstrated a robust interlayer structure and a continuous 3D electron transfer network, enabling efficient Zn^2+^ diffusion with low migration barriers and fast kinetics [98]. Its interlayer spacing of 10.7 Å, larger than that of VOH but smaller than that of K^+^ intercalated into VOH or PANI intercalated into VOH, results from strong bonding interactions between K^+^, oxygen, and nitrogen atoms, optimizing both stability and ion transport. The synergistic pillar effect of Mn^2+^ and PANI significantly improved the electron and ion transport kinetics in MnVO‐PANI, enabling a high capacity retention of 85% even after 10,000 cycles at a current density of 5 A g^−1^ [99].

Table 6 presents the interlayer spacing and electrochemical properties of V_2_O_5_ cathode materials cointercalated with metal ions and organic additives. Among these, [Al_0.16_(C_5_H_14_ON)_0.12_]V_2_O_5_·0.39H_2_O, owing to the synergistic combination of Al^3+^‐induced structural stabilization and organic molecule pillaring, which enlarges the interlayer spacing and facilitates rapid Zn^2+^ diffusion, exhibits the best specific capacity [97].

**TABLE 6:** Electrochemical performance and interlayer spacing of V_2_O_5_ cathode materials cointercalated with metal‐ion and organic additives.

Material	Capacity, mAh g^−1^	Capacity retention	Electrolyte	Interlayer spacing, Å	Voltage window, V	Synthesis method	Reference, year
PEG‐Ba_0.38_V_2_O_5_⋅nH_2_O	340.00 at 0.1 A g^−1^	98.80% after 4000 cycles at 6 A g^−1^	2 M ZnSO_4_	10.70	0.3–1.4	Hydrothermal and subsequent freeze‐drying	[92], 2022
FeVO‐12	408.00 at 0.1 A g^−1^	90.00% after 1000 cycles at 10 A g^−1^	2 M Zn(CF_3_SO_3_)_2_	19.29	0.3–1.6	Hydrothermal	[93], 2022
MgVOH/PANI	412.00 at 0.1 A g^−1^	93.00% after 1000 cycles at 2 A g^−1^	3 M Zn(CF_3_SO_3_)_2_	14.60	0.2–1.6	Hydrothermal	[94], 2024
ZBVO	417.10 at 0.1 A g^−1^	93.00% over 5000 cycles at 10 A g^−1^	3 M Zn(CF_3_SO_3_)_2_	13.50	0.3–1.6	Stirring	[95], 2024
(C_6_H_4_NH)_0.27_K_0.24_V_2_O_5_⋅0.92H_2_O	418.30 at 0.1 A g^−1^	89.50% after 3000 cycles at 5 A g^−1^	3 M Zn(CF_3_SO_3_)_2_	10.70	0.2–1.5	Hydrothermal and subsequent freeze‐drying	[98], 2024
Mn_0.18_V_2_O_5_⋅ 0.13H_2_O‐0.33PANI	462.00 at 0.1 A g^−1^	85.00% after 10 000 cycles at 5 A g^−1^	3 M ZnSO_4_	13.60	0.3–1.6	Hydrothermal	[99], 2022
([N(CH_3_)_4_]_0.77_,Zn_0.23_)V_8_O_20_· 3.8H_2_O	303.40 at 0.2 A g^−1^	99.50% after 2000 cycles at 8 A g^−1^	3 M Zn(CF_3_SO_3_)_2_	11.20	0.3–1.5	Hydrothermal	[96], 2021
[Al_0.16_(C_5_H_14_ON)_0.12_]V_2_O_5_· 0.39H_2_O	549.50 at 0.2 A g^−1^	80.10% after 20 000 cycles at 30 A g^−1^	3 M Zn(CF_3_SO_3_)_2_	13.70	0.2–1.6	Hydrothermal	[97], 2025

The synergistic strategy of combining organic molecules and inorganic‐ions, along with appropriate defects, improved the kinetic and structural stability of aqueous battery cathodes. Zong et al. [100] cointercalated metal ions (Zn^2+^) and organic‐ions (Ch^+^) into layered V_2_O_5_ to enhance its performance as a cathode material. This coinsertion strategy, combined with the introduction of oxygen vacancies, effectively expanded the interlayer spacing, increased the number of active sites for Zn^2+^ intercalation, and improved both electronic and ionic conductivity. In this system, Zn^2+^ ions act as structural pillars to stabilize the V–O layers, while the organic‐ions reduce electrostatic interactions, thereby accelerating reaction kinetics and enhancing structural stability.

Increasing interlayer spacing in V_2_O_5_ enhances capacity performance, but excessive expansion can destabilize the structure, leading to collapse during cycling and can reduce stability [96]. Additionally, side reactions at the cathode/electrolyte interface can cause vanadium dissolution, further compromising structural integrity and cycle life [101]. To address these issues, combining organic polymer coatings with V_2_O_5_ has proven effective. For example, CaVO/PANI composites‐formed by intercalating Ca^2+^ and applying a PANI coating‐enhanced structural stability, improved conductivity, and suppressed vanadium dissolution, achieving 233 mAh g^−1^ at 10 A g^−1^ and 93% capacity retention over 6000 cycles [102]. Similarly, Liu et al. [103] developed a La^3+^ and phytic acid‐modified V_2_O_5_ (LPVO) composite, where La^3+^ expanded interlayer spacing, and phytic acid coating reinforced structural stability and reduced vanadium loss during cycling. A highly promising strategy to further improve ZIB performance is to integrate metal‐ion intercalation, organic molecule pillaring, defect engineering, and surface polymer protection into a single hierarchical design.

### 3.2 Cointercalation of Metal Ions and Nonmetallic Species

Compared to metal ions, nonmetallic cations such as NH_4_^+^ and H^+^ possess lower molecular weights, thereby contributing to a higher theoretical capacity. However, incorporating a single type of interlayer cation may be insufficient to achieve optimal Zn^2+^ storage performance. To address this limitation, the introduction of mixed interlayer cations has emerged as a promising strategy to further enhance the electrochemical properties of hydrated V_2_O_5_. For instance, the coinsertion of Ni^2+^ and NH_4_^+^ into V_2_O_5_·3H_2_O not only expanded the interlayer spacing but also significantly improved Zn^2+^ transport kinetics [104]. Additionally, the synergistic “pillar” effect of Ni^2+^ and NH_4_^+^ increased oxygen vacancy concentration, effectively lowering the energy barrier for Zn^2+^ insertion. As a result, the electrode demonstrated a high specific capacity of 398.1 mAh g^−1^ at 1.0 A g^−1^ and excellent cycling stability, retaining 89.1% of its capacity after 2000 cycles at 5.0 A g^−1^. Moreover, a ternary hybrid cointercalated vanadium oxide cathode incorporating H_2_O, NH_4_^+^, and Na^+^ has been developed, which collaboratively enhanced the number of active Zn^2+^ storage sites, stabilized the lattice structure, and facilitated Zn^2+^ migration [105]. Owing to these synergistic effects, the cathode achieved near‐theoretical capacity at low current density and exhibited superior rate performance.

Heteroatom doping is also an effective strategy for introducing defects and enhancing the performance of vanadium‐based cathode materials. In 2023, Qian et al. [106] developed nitrogen‐doped, Mn preintercalated hydrated V_2_O_5_ (N‐MVO) via nitrogen plasma treatment. The Mn‐ions and strong V—N bonds stabilized the structure, while nitrogen‐induced oxygen vacancies weakened Zn^2+^–host interactions, improving rate capability and cycling stability. Another study reported partially nitrided, calcium‐doped vanadium oxide, in which nitrogen doping enhanced conductivity, and Ca^2+^ intercalation expanded the interlayer spacing [107]. This combination yielded a high capacity of 418.5 mAh g^−1^ at 0.1 A g^−1^ and 81.2% retention after 500 cycles at 2.0 A g^−1^. Chalcogenides like S^2−^, Se^2−^ can substitute lattice oxygen rather than intercalate, creating oxygen vacancies and V—S (V—Se) bonds that narrow the bandgap, enhance electronic conductivity, and improve redox kinetics. Introduction of halide anions such as F^−^ and Cl^−^ may also improve the electrochemical properties of vanadium oxide. F^−^ can substitute oxygen and create V—F bonds, which can increase the electronic conductivity and stabilize the structure. Cl^−^ occasionally intercalates electrostatically.

## 4 Multi‐Ion Intercalation

Recently, dual‐ or multi‐ion intercalation has been used to improve the overall performance of ZIBs in diverse application demands. Different metal ions interact uniquely with the vanadium oxide framework, resulting in varied impacts on its electrochemical behavior. Therefore, the coinsertion of heterovalent cations is anticipated to produce coordinated or synergistic enhancements in structural stability, ion transport, and capacity. Chen et al. [108] developed a Zn^2+^ and Mg^2+^ codoped V_2_O_5_ cathode that achieved 76.1% capacity retention after 6000 cycles at 10 A g^−1^. In this system, abundant Zn^2+^ ions enabled rapid Zn^2+^ storage and release during cycling, while preinserted Mg^2+^ acted as a structural pillar to expand the layer spacing, resulting in excellent cyclic stability.

**FIGURE 8:**
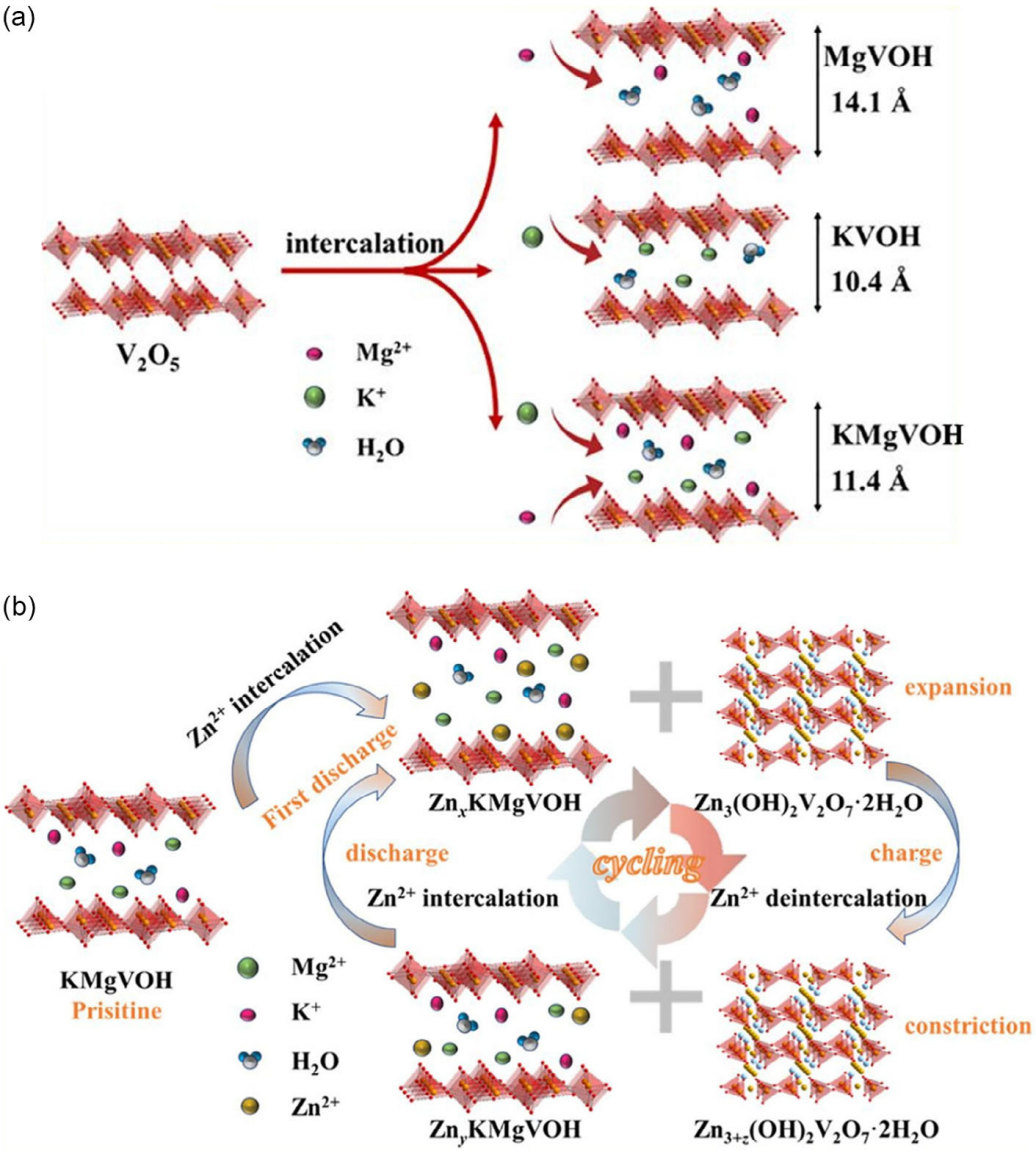
Effect of mixed intercalation and hydration. (a) MgVOH, KVOH, and KMgVOH (K_0.09_Mg_0.03_V_2_O_5_⋅nH_2_O) crystal structures and (b) schematic diagram of the Zn (de)intercalation mechanism in the K_0.09_Mg_0.03_V_2_O_5_⋅nH_2_O electrode, reproduced with permission [110], Copyright 2022, Elsevier.

Different from single metal‐ion insertion, the size of the inserted metal‐ions is not only the determining factor in altering the interlayer spacing for multi‐ions. The change in the interlayer spacing can also be altered by electrostatic attraction of the inserted metal ions with the framework oxygen [109, 110, 111]. K^+^ and Al^3+^ were simultaneously preinserted into vanadium oxide to form robust K_0.098_Al_0.12_V_2_O_5_⋅0.86H_2_O nano‐microspheres with a large interlayer spacing of 13.26 Å [109]. Although the ionic radius of K^+^ (1.38 Å) is higher than that of Al^3+^ (0.54 Å), the interlayer spacing of K^+^ into VOH (10.8 Å) is lower than that of Al^3+^ into VOH (14.3 Å). This is due to the higher ionic character of the K—O bond; it has a stronger binding energy, resulting in a lower interlayer spacing. Similarly, when Feng et al. [110] inserted Mg^2+^ and K^+^ into the VOH layer at the same time, the binding energies neutralized each other, resulting in a K_0.09_Mg_0.03_V_2_O_5_⋅nH_2_O layer spacing of 11.4 Å, identical to pristine VOH (Figure 8a). The Zn (de)intercalation mechanism in K_0.09_Mg_0.03_V_2_O_5_⋅nH_2_O, with both Mg^2+^ and K^+^ coinserted into the V–O interlayer, is illustrated in Figure 8b. Li et al. [111] coinserted the monovalent‐ion K^+^ and the divalent‐ion Ca^2+^ into VOH (K_0.02_Ca_0.18_V_2_O_5_⋅0.7H_2_O), its interlayer spacing is only slightly smaller than that of Ca^2+^ into VOH due to the relatively small amount of inserted K^+^ ions. This indicates that the quantity of inserted metal ions is closely related to the variation in interlayer spacing. Li et al. [112] introduced Ni^2+^ into the KVOH cathode material, where K^+^ is intercalated into VOH, to form a modified KNiVOH compound. The incorporation of Ni^2+^ enhanced the electronic conductivity and facilitated Zn^2+^ intercalation kinetics, while also stabilizing the K—O bond by reducing its formation energy. As a result, KNiVOH exhibited a higher specific capacity of 275 mAh g^−1^ at 5 A g^−1^, outperforming the original KVOH (266 mAh g^−1^).

Vanadium oxide nanobelts preintercalated with Na^+^ and Ca^2+^ were obtained via a simple solution impregnation method, and the synthesis strategy could be scaled up [113]. The presence of interlayer water molecules is inevitably introduced during the intercalation process of Na^+^ and Ca^2+^ ions. Although the presence of water molecules act as a “lubricant” to promote movement of Zn^2+^ ions, the presence of more water molecules can weaken the interlayers. By increasing the calcination temperature, the amount of interlayer water molecules decreases and the crystallinity of the nanobelt increases, but beyond a temperature of 400 °C, it shows cracks due to the structure shrinkage. TiK‐VOH, a bimetallic‐ion system with Ti^4+^ and K^+^ co‐preintercalated into VOH, features a lamellar, fish scale‐like structure synthesized using a simple sol–gel method [114]. The high charge density of Ti^4+^ enhances electron attraction within the V–O layers and modulates the interlayer spacing, thereby improving Zn^2+^ diffusion and structural stability. Simultaneously, K^+^ boosts electrical conductivity and accelerates charge transfer during electrochemical reactions. Jiang et al. [115] developed a “dual‐ion‐in‐sequence” intercalation strategy, successfully implemented through thermal quenching, to synthesize the Li@MnVO cathode material. Li@MnVO have Mn‐ and Li‐ions intercalation together with abundant defects formed on the surface/subsurface.

Electrochemical performance and interlayer spacing of multi‐ion intercalated vanadium peroxide cathode materials are summarized in Table 7. The high specific capacity of NCVO‐350 is attributed to the preintercalated bimettalic‐ions within the layers that was generally added to enhance the structural stability of vanadium oxide [113].

**TABLE 7:** Electrochemical performance and interlayer spacing of multi‐ion intercalated V_2_O_5_ cathode materials.

Material	Capacity, mAh g^−1^	Capacity retention	Electrolyte	Interlayer spacing, Å	Voltage window, V	Synthesis method	Reference, year
K_0.098_Al_0.12_V_2_O_5_⋅0.86H_2_O	424.00 at 0.05 A g^−1^	96.00% after 3000 cycles at 5 A g^−1^	3 M Zn(CF_3_SO_3_)_2_	13.26	0.2–1.6	Hydrothermal	[109], 2024
K_0.09_Mg_0.03_V_2_O_5_⋅nH_2_O	423.00 at 0.1 A g^−1^	222 mAh g^−1^ after 2000 cycles at 4 A g^−1^	3 M Zn(CF_3_SO_3_)_2_	11.40	0.2–1.5	Hydrothermal	[110], 2022
TiK‐VOH	393.40 at 0.2 A g^−1^	94.00% after 2000 cycles	3 M Zn(CF_3_SO_3_)_2_	12.10	0.3–1.6	Sol–gel	[114], 2024
(Zn_0.09_,Mg_0.09_)V_2_O_5_·0.81H_2_O	~450.00 at 0.2 A g^−1^	76.10% after 6000 cycles at 10 A g^−1^	2 M Zn(CF_3_SO_3_)_2_	12.40	0.2–1.8	Hydrothermal	[108], 2025
K_0.02_Ca_0.18_V_2_O_5_⋅0.7H_2_O	410.00 at 0.3 A g^−1^	265 mAh g^−1^ after 1800 cycles at 5 A g^−1^	3 M Zn(CF_3_SO_3_)_2_		0.3–1.5	Hydrothermal	[111], 2024
NCVO‐350	310.00 at 0.5 A g^−1^	100.00% after 200 cycles 0.5 A g^−1^	2 M Zn(CF_3_SO_3_)_2_		0.2–1.6	Solution impregnation and subsequent freeze‐drying	[113], 2022
KNiVOH	275.00 at 5 A g^−1^		3 M Zn(CF_3_SO_3_)_2_	11.00	0.2–1.6	Hydrothermal	[112], 2022

Ma et al. [116] successfully synthesized a tri‐metal‐ion intercalated cathode material (KMgAl‐VOHs) via a simple one‐step hydrothermal method by doping alkali metal‐ion K^+^, alkaline earth metal‐ion Mg^2+^, and trivalent metal‐ion Al^3+^. Benefiting from the synergistic effect of these preintercalated ions, the optimized tri‐metal‐ion hydrated V_2_O_5_ cathode delivers a high specific capacity of 382.4 mAh g^−1^ at 0.5 A g^−1^ and demonstrates excellent long‐term cycling stability with 86% capacity retention after 2000 cycles at a high current density of 10 A g^−1^. By adjusting the relative proportions of monovalent, divalent, and trivalent metal ions, which differ in electronegativity and ion size, an optimal interlayer spacing for a stable structure and a uniform morphology can be achieved to enhance Zn^2+^ transport kinetics. With the increase of K^+^ ions and the decrease of Mg^2+^, the interlayer space decreases gradually. The size of microspheres gradually increases with increase of intercalated K^+^, whereas the nanowires on the surface of KMgAl‐VOHs microspheres narrows. Also the embedding of Mg^2+^ and Al^3+^ results in maintaining a rough surface of the microsphere and thinner surface nanostructures, respectively.

Based on the summarized results, another promising strategy to enhance battery performance would be the design of a multi‐ion preintercalated hydrated V_2_O_5_ system that combines high charge density cations with conductivity while precisely regulating interlayer water content through controlled thermal treatment. The insertion of monovalent metal ions can slightly reduce the VO layer spacing and play a supporting role between the layers to stabilize the framework. Divalent metal ions effectively expand the lattice spacing and enhance the electrical conductivity and Zn^2+^ diffusion. Moreover, trivalent metal ions are beneficial for buffering lattice volume changes during cycling and increasing the proportion of V^4+^. However, the underlying mechanisms responsible for the performance enhancement in multi‐ion engineering strategies are still not fully understood and warrants further systematic investigation.

## 5 Design Principles for Preintercalated V_2_O_5_ Cathodes

Building on the mechanistic understanding established throughout this review, preintercalation in V_2_O_5_ can be rationalized as a coupled structural–electrostatic design problem, rather than a purely compositional modification. The performance of preintercalated V_2_O_5_ cathodes emerges from the interplay between interlayer geometry, electrostatic screening, and redox‐active lattice dynamics. From this perspective, the following design principles can be defined.

### 5.1 Interlayer Spacing as a Tunable Transport Parameter

The interlayer distance is the primary structural descriptor governing Zn^2+^ diffusion. Optimal performance is not achieved by maximizing spacing, but by balancing accessibility and structural coherence. Excessive expansion weakens interlayer interactions and promotes structural collapse, whereas insufficient spacing leads to strong electrostatic trapping of Zn^2+^.

Preintercalated species should therefore act as mechanically robust pillars that maintain an intermediate interlayer spacing (typically ∼10–13 Å in hydrated systems), enabling fast ion transport while preserving long‐term cycling stability. As highlighted earlier, this spacing is codetermined by the ionic radius, hydration shell, and concentration of the intercalant. The “intermediate interlayer spacing” refers to an empirically observed spacing window rather than a theoretically optimized value. Based on the hydrated and preintercalated V_2_O_5_‐based cathodes summarized in this review, many high‐performing systems exhibit interlayer distances in the approximate range of 10–13 Å. This range reflects a balance between two competing effects: insufficient spacing strengthens steric confinement and Zn^2+^–host electrostatic interactions, whereas excessive expansion may weaken interlayer cohesion and compromise structural stability. Therefore, the ∼10–13 Å range should be considered a practical experimental guideline for hydrated V_2_O_5_ systems, not a universal structural requirement for all layered oxides.

### 5.2 Electrostatic Screening and Effective Charge Reduction

A defining feature of aqueous Zn^2+^ systems is the strong Coulombic interaction between divalent ions and the oxide lattice. Effective design requires modulating the local electrostatic potential landscape. Preintercalated ions and structural water jointly act as electrostatic mediators, reducing the effective charge of Zn^2+^ through: **(1)** dielectric screening; **(2)** solvation effects; and **(3)** charge redistribution within the lattice. This results in lower diffusion barriers and improved reversibility, particularly in hydrated V_2_O_5_ phases where water behaves as an active electrostatic component rather than a passive spacer.

### 5.3 Charge Density and Ionic Size Matching

**FIGURE 9:**
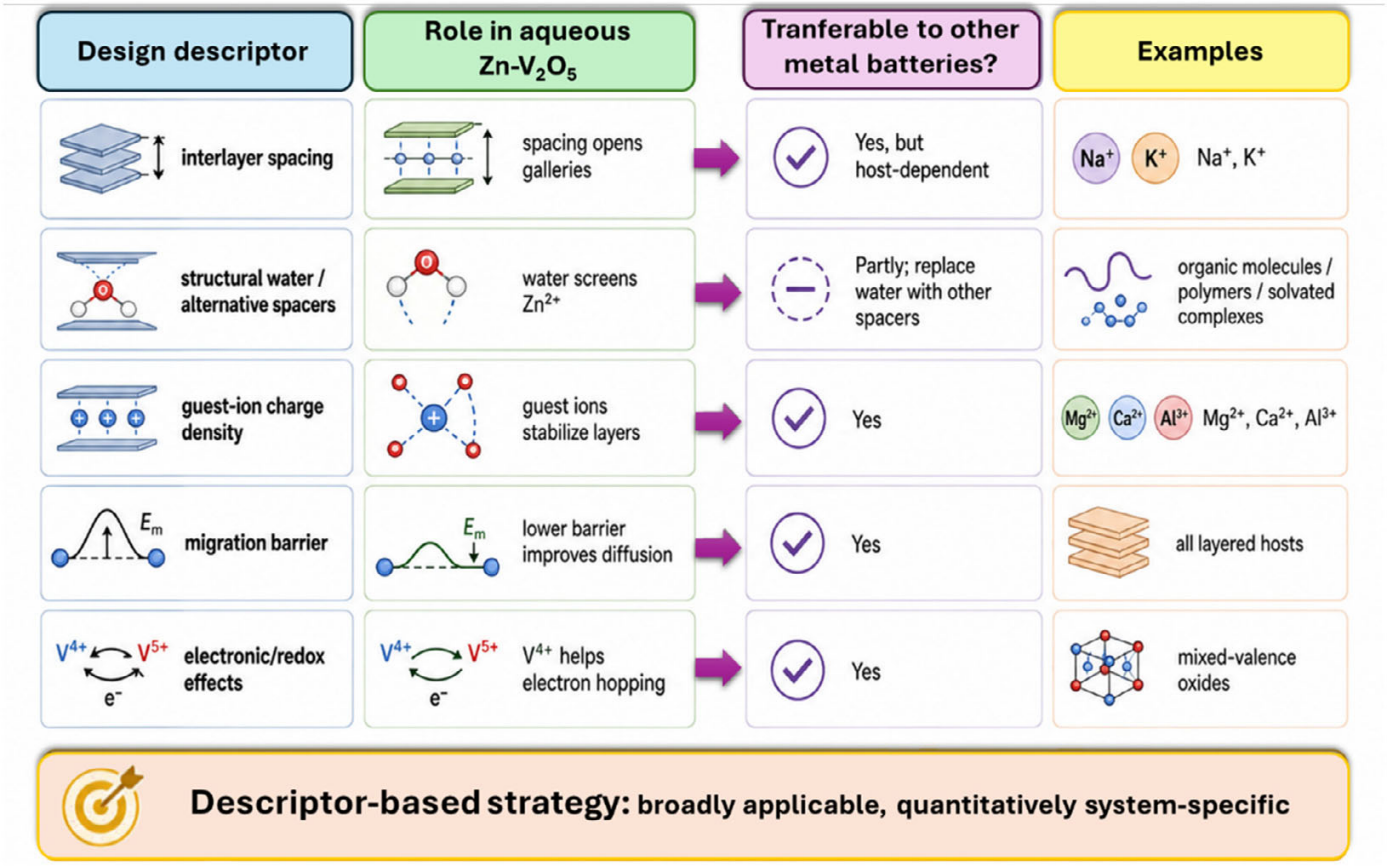
Transferability of preintercalation design principles beyond V_2_O_5_‐based aqueous ZIBs. The schematic illustrates how the descriptor‐based framework established for hydrated V_2_O_5_ cathodes—including interlayer spacing regulation, electrostatic screening, guest‐ion charge density/pillaring, migration‐barrier reduction, and improved electronic transport—can be extended to other layered or open‐framework metal‐battery systems. These principles remain relevant for monovalent systems (Na^+^, K^+^), multivalent systems (Mg^2+^, Ca^2+^), and high‐charge or hybrid‐ion systems (e.g., Al^3+^), although their relative importance and optimal values depend on the host structure, electrolyte chemistry, and solvation/desolvation environment. The figure highlights that the framework is transferable but not universal, providing generalizable descriptors while requiring system‐specific optimization.

The selection of preintercalated species must consider both ionic radius and charge density, as these jointly determine interaction strength with the host lattice (Figure 9): **(1)** monovalent metal ions (e.g., K^+^) provide weak electrostatic interaction, facilitating Zn^2+^ mobility and enhancing rate capability; **(2)** divalent metal ions (e.g., Ca^2+^, Mn^2+^, Cu^2+^) introduce stronger binding, improving structural stability but potentially increasing diffusion barriers; and **(3)** oversized monovalent metal ions (e.g., Cs^+^) may stabilize the structure but hinder ion transport due to steric effects. Thus, optimal performance arises from intermediate charge density and size, where structural stabilization does not compromise ion kinetics.

### 5.4 Synergistic Role of Structural Water

Structural water is a critical, nontrivial design parameter. Rather than being treated as a byproduct of synthesis, it should be deliberately engineered. Water molecules: **(1)** expand interlayer spacing; **(2)** provide additional coordination sites; **(3)** reduce Zn^2+^–lattice interaction strength; and **(4)** homogenize the electrostatic field. However, excess water weakens interlayer cohesion, while insufficient water increases diffusion barriers. Therefore, an optimal hydration level must be maintained, enabling a “lubricated” diffusion environment without structural degradation.

### 5.5 Electronic Structure and Mixed Valence Control

Preintercalation modifies not only the structure but also the electronic landscape of V_2_O_5_, particularly through the V^5+^/V^4+^ ratio. An increased V^4+^ content: **(1)** boosts electronic conductivity; **(2)** promotes electron hopping; and **(3)** enhances redox kinetics. Thus, effective design requires coupling ionic and electronic transport, ensuring that improvements in Zn^2+^ mobility are matched by sufficient electronic conductivity.

### 5.6 Occupancy Versus Accessibility

A critical limitation of preintercalation is the partial occupation of active sites. While intercalants stabilize the structure, excessive loading reduces the number of available Zn^2+^ storage sites.

This leads to a fundamental tradeoff: **(1)** higher intercalant concentration → better stability and **(2)** lower intercalant concentration → higher capacity. Optimal systems therefore operate in a substoichiometric regime, where interlayer stabilization is achieved without significantly compromising storage capacity.

### 5.7 Multicomponent and Cooperative Intercalation

The highest‐performing systems often arise from synergistic multispecies intercalation, where: **(1)** metal ions provide structural rigidity; **(2)** water molecules enable electrostatic screening; and **(3)** organic or polymeric species tune spacing and flexibility. Such cooperative effects generate emergent behavior not accessible through single‐component systems, including enhanced cycling stability, rate capability, and tolerance to structural distortion.

### 5.8 Toward a Unified Design Map

These principles collectively define a multidimensional design space, where performance is governed by the coupling between: **(1)** interlayer spacing; **(2)** electrostatic environment; **(3)** ionic size and charge; **(4)** hydration level; and **(5)** electronic structure. Rather than optimizing individual parameters independently, future strategies should adopt a holistic design approach, targeting the simultaneous control of structural and electrostatic variables (Figure 9). Preintercalation operates through a coupled structural–electrostatic–solvation mechanism.

To clarify the generality of this mechanism, preintercalation can be described using a simple descriptor‐based framework rather than a material‐specific rule. In layered V_2_O_5_ and related oxides, the ease of Zn^2+^ or multivalent‐ion migration is governed by the balance between interlayer opening, electrostatic screening, lattice stabilization, and preservation of accessible storage sites. This relationship can be qualitatively expressed as

(1) $$E_{a}\;∼\;f\left(d_{\text{inter}},\;z_{\text{eff}},\;\varepsilon_{\mathrm{loc}},n_{H_{2}O},\;K_{\text{layer}},\text{Δ}\phi_{\text{dist}},\;\theta_{\text{guest}},\sigma_{e}\right)$$

where $E_{a}$ is the apparent ion‐migration barrier, $d_{\text{inter}}$ is the interlayer spacing, $z_{\mathrm{eff}}$ is the effective charge of the migrating ion after solvation and screening, $\varepsilon_{\mathrm{loc}}$ reflects the local dielectric/electrostatic screening environment, $n_{H_{2}O}$ represents the contribution of structural water, $K_{\text{layer}}$ describes the mechanical rigidity of the oxide layers, $\text{Δ}\phi_{\text{dist}}$ represents the distortion or excessive structural constraint, $\theta_{\text{guest}}$ represents the fraction of interlayer sites occupied by preintercalated species, and $\sigma_{e}$ is the electronic conductivity or redox accessibility of the host.

Within this framework, preintercalation improves ion storage when it expands or stabilizes the interlayer gallery, screens the strong electrostatic interaction between multivalent ions and the oxide framework, and suppresses irreversible layer collapse during cycling. However, excessive or strongly bound guest species may reduce performance by occupying active sites, increasing local migration resistance, or inducing lattice distortion. Therefore, the optimum preintercalated structure is not necessarily the one with the largest interlayer spacing, but the one that achieves the best compromise between spacing, electrostatic screening, structural rigidity, hydration, and site accessibility. The phenomenological expansion is as follows

(2) $$E_{a}=E_{0}-\alpha \;d_{\text{inter}}-\beta \;\varepsilon_{\mathrm{loc}}-\gamma \;n_{H_{2}O}-\delta \;\sigma_{e}+\lambda \;\frac{z_{\mathrm{eff}}^{2}}{\;\varepsilon_{\mathrm{loc}}}-\eta \;K_{\text{layer}}+\mu \;\theta_{\text{guest}}+\nu \;\text{Δ}\phi_{\mathrm{dist}}$$

The diffusion equation is as follows

(3) $$D_{\mathrm{ion}}=D_{0}e^{-\frac{E_{a}}{k_{B}T}}=D_{0}e^{-\frac{E_{0}-\alpha \;d_{\text{inter}}-\beta \;\varepsilon_{\mathrm{loc}}-\gamma \;n_{H_{2}O}-\delta \;\sigma_{e}-\eta \;K_{\text{layer}}+\lambda \;\frac{z_{\mathrm{eff}}^{2}}{\;\varepsilon_{\mathrm{loc}}}+\mu \;\theta_{\text{guest}}+\nu \text{Δ}\phi_{\mathrm{dist}}}{k_{B}T}}$$

where $E_{0}$ is the unmodified apparent ion‐migration barrier. We have written the model in terms of $\frac{z_{\text{eff}}^{2}}{\;\varepsilon_{\text{loc}}}$, rather than simply $z_{\text{eff}}^{2}$, because the electrostatic contribution to ion trapping, desolvation, and polarization scales approximately with the square of the effective ionic charge and is reduced by the local dielectric/electrostatic screening environment. Thus, structural water and preintercalated species influence transport not only by expanding the interlayer spacing, but also by lowering the effective electrostatic penalty for Zn^2+^ migration.

This descriptor‐based diffusion model, as in Equations (1) – (3), also explains why different guest species lead to different electrochemical outcomes. Monovalent metal ions generally provide moderate electrostatic interaction and efficient spacing regulation; divalent metal ions can offer stronger lattice stabilization but may increase ion–host interaction strength; trivalent metal ions or highly polarizing ions can further stabilize the framework but require careful control to avoid excessive trapping or site blocking. Structural water acts as an additional electrostatic mediator by weakening Zn^2+^–host interactions and facilitating smoother ion transport. These descriptors are common to other layered oxide cathodes, including hydrated manganese oxides, molybdenum oxides, and mixed transition‐metal oxides, suggesting that the framework can guide preintercalation design beyond V_2_O_5_ based zinc‐ion systems.

Structural water should therefore be regarded as a particularly effective electrostatic mediator in hydrated V_2_O_5_‐based cathodes, but not as a universally transferable requirement for all layered oxide systems. Its beneficial role is most evident in aqueous ZIBs, where interlayer water simultaneously expands the gallery spacing, weakens Zn^2+^‐host electrostatic interactions, participates in hydrogen‐bond‐assisted transport, and reduces the effective ion‐migration penalty. In nonaqueous or low‐water‐content systems, these functions may be significantly reduced or absent, and Zn^2+^ transport may depend more strongly on lattice channels, host—guest bonding, and the desolvation environment imposed by the electrolyte. Therefore, the structural‐water mechanism should not be directly extrapolated to water‐deficient systems without further experimental or computational validation. Instead, similar electrostatic‐screening and lattice‐stabilizing effects may need to be introduced through alternative interlayer species, such as organic molecules, polymer pillars, ionic‐liquid‐derived species, or solvated metal‐ion complexes. This represents an important direction for extending preintercalation design principles beyond hydrated V_2_O_5_ and aqueous ZIB systems. Related oxide‐interface and solid–electrolyte studies further support this broader view by showing that oxide work functions, interfacial electrochemistry, and the distinction between dielectric oxides and solid electrolytes are central to energy‐storage performance [117, 118, 119]. Recent studies on inorganic–organic cointercalated vanadates, polyaniline‐intercalated vanadium oxides, alcohol‐molecule‐coupled aqueous zinc‐ion cathodes, and organic‐intercalation‐engineered V_2_CT_*x*_ MXene further illustrate that alternative organic, polymeric, or molecular spacers can regulate interlayer chemistry, ion migration, and structural stability beyond simple hydrated V_2_O_5_ systems [120, 121, 122, 123].

A comparative perspective among the main modification strategies highlights that no single approach simultaneously optimizes cost, structural stability, and electrochemical performance (Table 8). Simple metal‐ion preintercalation is generally the most cost‐effective and scalable route, as it commonly relies on hydrothermal or stirring methods using relatively inexpensive alkali or alkaline‐earth salts. Its main advantage is the stabilization of the V_2_O_5_ interlayer galleries through pillaring and electrostatic regulation, although excessive guest‐ion loading may block active Zn^2+^ storage sites or introduce lattice strain. Defect engineering provides a more direct route to tune electronic structure, increase active sites, and accelerate Zn^2+^ diffusion through oxygen vacancies, vanadium vacancies, or heteroatom‐induced charge redistribution. However, it requires more precise control of defect concentration, since excessive defects may compromise long‐range structural integrity. Carbon composite strategies are particularly effective in overcoming the low intrinsic electronic conductivity of vanadium oxides, while also buffering mechanical stress, suppressing particle aggregation, and improving high‐rate performance. Their main limitation is the greater processing complexity associated with conductive additive integration, freeze‐drying, annealing, or multistep composite formation. Therefore, in terms of cost and scalability, the approximate ranking is preintercalation > defect engineering > carbon composites; in terms of electronic/kinetic enhancement, carbon composites and defect engineering generally outperform preintercalation alone; and in terms of balanced overall performance, hybrid architectures that combine controlled preintercalation, moderate defect engineering, and conductive carbon networks are the most promising.

**TABLE 8:** Comparative ranking of V_2_O_5_ modification strategies in aqueous Zn‐ion battery cathodes.

Strategy	Cost/Scalability	Structural stability	Electrochemical performance	Main limitation	Representative recent references
Metal‐ion preintercalation	High	High, through interlayer pillaring	Moderate to high	May not fully solve poor electronic conductivity	[38, 51, 52, 53, 54, 55, 56, 57, 58, 59, 60, 61, 62, 63, 64, 65, 66, 67, 68, 69, 70, 71, 72, 73, 74, 75, 108, 109, 110, 111, 112, 113, 114]
Defect engineering	Moderate	Moderate to high if defect density is controlled	High, due to improved kinetics and active sites	Excess defects may destabilize the lattice	[85, 86, 87, 88, 89, 90, 91]
Carbon composites	Moderate to low	High, due to mechanical buffering	High, especially at high rate	More complex processing	[76, 77, 78, 79, 80, 81, 82, 83, 84]
Hybrid strategies	Moderate	Very high	Highest overall balance	Requires careful synthetic optimization	[91, 92, 93, 94, 95, 96, 97, 98, 99, 100, 101, 102, 103, 108, 109, 110, 111, 112, 113, 114]

## 6 Conclusion and Perspectives

Metal ion preintercalation is an effective strategy in addressing structural instability and limited interlayer spacing in V_2_O_5_ which also enhance overall electrochemical performance of aqueous ZIBs. Structural water plays a pivotal role in regulating the crystal structure and electrochemical properties of V_2_O_5_⋅nH_2_O and related M_*x*_V_2_O_5_·nH_2_O cathodes for aqueous ZIBs. When combined with preintercalated metal ions, structural water synergistically boosts electronic conductivity, Zn^2+^ transport, and mitigates structural degradation during long‐term cycling. However, excessive hydration leads to weakened interlayer bonding and structural instability.

The insertion of monovalent metal ions can slightly contract the V_2_O_5_ layer spacing while acting as interlayer supports. In contrast, divalent metal ions expand the lattice spacing; enhance the electrical conductivity and Zn^2+^ diffusion. Moreover, trivalent metal ions help buffer lattice volume changes during cycling and increase the V^4+^ content. Hydrated metal‐ion intercalation primarily enhances structural stability and alleviates electrostatic repulsion, while cointercalation of metal ions and water fundamentally modifies the charge‐storage mechanism by introducing fast proton kinetics and synergistic dual‐carrier transport, resulting in higher capacity, superior rate capability, and enhanced cycling stability. However, too many preintercalated ions can occupy the active Zn^2+^ storage sites and reduce the capacity and battery performance. The integration of conductive materials addresses the intrinsic low conductivity of vanadium oxide. It enhances ion transport, reduces polarization resistance, and mitigates structural degradation during cycling. Moreover, the introduction of oxygen vacancies and cationic defects has proven effective in enhancing electronic conductivity, active intercalation sites and promoting faster ion migration. The combined strategy of metal‐ion intercalation and these engineering has led to significant improvements in specific capacity and long‐term cycling stability, as reported in several studies.

The cointercalation of metal ions and organic additives into V_2_O_5_ cathodes synergistically augments structural stability, ion diffusion, and specific capacity. Organic additives increase interlayer spacing, facilitate Zn^2+^ transport and increase active sites, while metal ions stabilize the structure through electrostatic interactions. This combination boosts ionic and electronic conductivity, suppresses vanadium dissolution, and enhances cycling stability by mitigating side reactions at the cathode/electrolyte interface. The introduction of mixed interlayer cations has emerged as a promising strategy to further enhance the electrochemical properties of V_2_O_5_. The insertion of multiple metal ions with different valence states is anticipated to produce coordinated or synergistic enhancements in structural stability, ion transport, and capacity. By adjusting the relative proportions of monovalent, divalent, and trivalent metal ions, which differ in electronegativity and hydrated ion size, an optimal interlayer spacing, a stable structure, and a uniform morphology can be achieved, thereby enhancing Zn^2+^ transport kinetics. Cointercalation of metal ions with MXene into hydrated vanadium oxide can make a better cathode material for ZIBs. 2D MXene, which has a flexible, hydrophilic surface with a layered structure similar to that of layered V_2_O_5_, can act as a conductive highway with a strong mechanical spacer that can be an antirestacking agent.

While cointercalation ions or molecules have different physicochemical properties, their functional mechanisms are categorized into three primary mechanisms. First, ions tend to occupy specific sites within the host structures and act as pillars between V_2_O_5_ layers, thereby widening the interlayer spacing and enhancing structural stability. Second, the presence of high‐valence metal ions or larger molecules between the V_2_O_5_ layers helps shield electrostatic interactions between Zn^2+^ ions and the VO framework. This shielding enhances Zn^2+^ diffusion kinetics, allowing better accessibility of Zn^2+^ ions to the vanadium oxide structure. Finally, the intercalated ions or molecules provide additional storage sites for Zn^2+^, a mechanism particularly observed in organic‐intercalated and defect‐engineered vanadium oxides.

The reversible cointercalation of solvated molecules in preintercalated cathodes of aqueous ZIBs has been reported in the literature. However, their effects and underlying mechanisms remain unclear, particularly regarding their impact on structural stability after prolonged cycling. Therefore, a deeper understanding of the role of guest‐ions or molecules influencing redox behavior within the battery system is still required. There is still a general lack of understanding regarding the optimal amount of crystal water needed to achieve enhanced electrochemical performance. Additionally, an excessive amount of structural water can expand the interlayer spacing of vanadium oxides, leading to the dissolution of active materials and the eventual collapse of the layered structure. Thus, it is crucial to determine the precise balance between structural stability and rapid ion kinetics. Since both anion and cation defects can improve electrochemical performance, it is important to find new ways to create vanadium oxide cathodes with precise control over the types, amounts of defects, where these defects are located and how they move within the vanadium oxide structure during repeated cycling.

Some of the major directions that can significantly shape the next generation of V_2_O_5_‐based cathode materials in these aspects are atomic‐level in operando characterization using XRD, XPS, TEM, and synchrotron‐based XAS to understand the interaction of metal ions with V_2_O_5_ layers during intercalation, cycling, and redox processes through ion migration pathways. Further, integrating computational tools such as DFT with molecular dynamics (MD) to simulate long‐term stability and Zn^2+^ diffusion kinetics along with machine learning (ML) algorithms to predict optimal intercalants, concentration windows, and synergistic combinations.

The instability of vanadium compounds in certain electrolytes remains a major drawback, including low ionic conductivity in organic electrolytes, a higher freezing point (about −10 °C) in aqueous electrolytes, and dendrite formation in alkaline electrolytes. However, using a neutral or low‐acidic electrolyte, a solid electrolyte, or a solvent mixture could potentially resolve these issues. High concentration electrolytes are also ideal for inhibiting the dissolution of electrode materials and broadening the voltage window of aqueous ZIBs but they often cause slow diffusion kinetics, leading to unsatisfactory capacity and rate performance. Therefore, it is important to develop a high‐performance electrolyte that is compatible with high performance electrode material. It is widely agreed that controlling the microstructure and texture of vanadium oxide particles is key to improving their electrochemical performance. However, several lab‐scale synthesis methods are difficult to scale up for commercial productions. At the same time, creating nanostructures or nanoparticles with larger surface area for electrolyte exposure can increase specific capacity through pseudocapacitive effects.

These advancements emphasize the value of synergistic design approaches in the development of high‐performance energy storage materials. With ongoing exploration of novel material combinations and fabrication techniques, vanadium oxide‐based composites hold strong promise for next‐generation aqueous ZIBs and other sustainable energy storage technologies.

## Author Contributions

**Anagha P. Vincent:** writing – original draft, conceptualization, literature review, formal analysis. **P. Vipin Kumar:** writing – original draft, conceptualization, literature review, formal analysis. **S. B. Gudennavar:** writing – review and editing, supervision, resources. **B. S. Nishchith:** writing – review and editing, supervision, resources. **S. G. Bubbly:** conceptualization, writing – review and editing, supervision, resources. **Maria Helena Braga**: writing – review and editing, visualization, funding acquisition, supervision, resources.

## Funding

This study was supported by CHRIST (Deemed to be University) (Grant CU‐ORS‐SM‐24/19), Government of India, New Delhi (Grant DST/INSPIRE Fellowship/2024/IF240147), PRR ‐ Recovery and Resilience Plan and the European Union's NextGeneration EU funds (Grant C644936001‐00000045), Fundação para a Ciência e a Tecnologia (FCT) (Grant UID/50022/2025 – Thematic line 6), and Universidade do Porto.

## Conflicts of Interest

The authors declare no conflicts of interest.

## Data Availability

Data sharing not applicable to this article as no datasets were generated or analyzed during the current study.

## References

[1] W. Ling , P. Wang , Z. Chen , et al., “Nanostructure Design Strategies for Aqueous Zinc‐Ion Batteries,” ChemElectroChem 7 (2020): 2957–2978.

[2] B. Tang , L. Shan , S. Liang , and J. Zhou , “Issues and Opportunities Facing Aqueous Zinc‐Ion Batteries,” Energy & Environmental Science 12 (2019): 3288–3304.

[3] L. E. Blanc , D. Kundu , and L. F. Nazar , “Scientific Challenges for the Implementation of Zn‐Ion Batteries,” Joule 4 (2020): 771–799.

[4] H. Pan , Y. Shao , P. Yan , et al., “Reversible Aqueous Zinc/Manganese Oxide Energy Storage from Conversion Reactions,” Nature Energy 1 (2016): 1–7.

[5] G. Li , Z. Yang , Y. Jiang , et al., “Towards Polyvalent Ion Batteries: A Zinc‐Ion Battery Based on NASICON Structured Na_3_V_2_(PO_4_)_3_ ,” Nano Energy 25 (2016): 211–217.

[6] N. Zhang , F. Cheng , J. Liu , et al., “Rechargeable Aqueous Zinc‐Manganese Dioxide Batteries with High Energy and Power Densities,” Nature Communications 8 (2017): 1–9. 10.1038/s41467-017-00467-x PMC5581336 28864823

[7] H. Glatz , E. Lizundia , F. Pacifico , and D. Kundu , “An Organic Cathode Based Dual‐Ion Aqueous Zinc Battery Enabled by a Cellulose Membrane,” ACS Applied Energy Materials 2 (2019): 1288–1294.

[8] J. Ming , J. Guo , C. Xia , W. Wang , and H. N. Alshareef , “Zinc‐Ion Batteries: Materials, Mechanisms, and Applications,” Materials Science and Engineering: R: Reports 135 (2019): 58–84.

[9] C. Xu , B. Li , H. Du , and F. Kang , “Energetic Zinc Ion Chemistry: The Rechargeable Zinc Ion Battery,” Angewandte Chemie International Edition 51 (2012): 933–935. 22170816 10.1002/anie.201106307

[10] N. Zhang , X. Chen , M. Yu , Z. Niu , F. Cheng , and J. Chen , “Materials Chemistry for Rechargeable Zinc‐Ion Batteries,” Chemical Society Reviews 49 (2020): 4203–4219. 32478772 10.1039/c9cs00349e

[11] X. Zhang , J. P. Hu , N. Fu , et al., “Comprehensive Review on Zinc‐ion Battery Anode: Challenges and Strategies,” InfoMat 4 (2022): e12306.

[12] J. R. Loh , J. Xue , and W. S. V. Lee , “Challenges and Strategies in the Development of Zinc‐Ion Batteries,” Small Methods 7 (2023): 2300101. 10.1002/smtd.202300101 37035953

[13] G. Li , L. Sun , S. Zhang , et al., “Developing Cathode Materials for Aqueous Zinc Ion Batteries: Challenges and Practical Prospects,” Advanced Functional Materials 34 (2024): 2301291.

[14] J. Kumankuma‐Sarpong , W. Guo , and Y. Fu , “Advances of Metal Oxide Composite Cathodes for Aqueous Zinc‐Ion Batteries,” Advanced Energy and Sustainability Research 3 (2022): 2100220.

[15] M. H. Alfaruqi , V. Mathew , J. Gim , et al., “Electrochemically Induced Structural Transformation in a γ‐MnO_2_ Cathode of a High Capacity Zinc‐Ion Battery System,” Chemistry of Materials 27 (2015): 3609–3620.

[16] A. Guo , Z. Wang , L. Chen , et al., “A Comprehensive Review of the Mechanism and Modification Strategies of V_2_O_5_ Cathodes for Aqueous Zinc‐Ion Batteries,” ACS Nano 18 (2024): 27261–27286. 39319501 10.1021/acsnano.4c09899

[17] Q. He , T. Hu , Q. Wu , et al., “Tunnel‐Oriented VO_2_ (B) Cathode for High‐Rate Aqueous Zinc‐Ion Batteries,” Advanced Materials 36 (2024): 2400888. 10.1002/adma.202400888 38490965

[18] F. Liang , Z. Zou , R. Zheng , Z. Xie , L. Song , and Y. Wang , “Crystalline Water Pre‐Embedding and Defect Engineering Endows V_6_O_13_ with Enhanced Zn²^+^ Ion Storage,” Chemical Engineering Journal. 522 (2025): 168100.

[19] M. Yan , P. He , Y. Chen , et al., “Water‐Lubricated Intercalation in V_2_O_5_ ·nH_2_O for High‐Capacity and High‐Rate Aqueous Rechargeable Zinc Batteries,” Advanced Materials 30 (2018): 1703725. 10.1002/adma.201703725 29131432

[20] O. D. Saliu , O. Iresemowo , F. Kubi , K. H. Moberuagba , A. G. Adeniyi , and J. Ramontja , “Enhancing the Performance of MoO_3_‐Based Cathodes for Aqueous Zinc‐Ion Batteries: Spinel CuMoO_3_ vs. Non‐Spinel CuO–MoO_3_ ,” Journal of Materials Science 20 (2025): 72.

[21] X. Zhou , P. Cao , A. Wei , et al., “Driving the Interfacial Ion‐Transfer Kinetics by Mesoporous TiO_2_ Spheres for High‐Performance Aqueous Zn‐Ion Batteries,” ACS Applied Materials & Interfaces 13 (2021): 8181–8190. 33560817 10.1021/acsami.0c18433

[22] W. Sun , F. Wang , S. Hou , et al., “Zn/MnO_2_ Battery Chemistry With H^+^ and Zn^2+^ Coinsertion,” Journal of the American Chemical Society 139 (2017): 9775–9778. 28704997 10.1021/jacs.7b04471

[23] Z. Liu , G. Pulletikurthi , and F. Endres , “A Prussian Blue/Zinc Secondary Battery with a Bio‐Ionic Liquid–Water Mixture as Electrolyte,” ACS Applied Materials & Interfaces 8 (2016): 12158–12164. 27119430 10.1021/acsami.6b01592

[24] M. Iturrondobeitia , O. Akizu‐Gardoki , O. Amondarain , R. Minguez , and E. Lizundia , “Environmental Impacts of Aqueous Zinc Ion Batteries Based on Life Cycle Assessment,” Advanced Sustainable Systems 6 (2022): 2100308.

[25] J. Sun , Y. Zhang , Y. Liu , et al., “Hydrated Vanadium Pentoxide/Reduced Graphene Oxide‐Polyvinyl Alcohol (V_2_O_5_⋅nH_2_O/rGO‐PVA) Film as a Binder‐Free Electrode for Solid‐State Zn‐Ion Batteries,” Journal of Colloid and Interface Science 587 (2021): 845–854. 33256962 10.1016/j.jcis.2020.10.148

[26] T. Zhou , Q. Han , L. Xie , X. Yang , L. Zhu , and X. Cao , “Recent Developments and Challenges of Vanadium Oxides (V_x_O_y_) Cathodes for Aqueous Zinc‐Ion Batteries,” Chemical Record 22 (2022): e202100275. 34962053 10.1002/tcr.202100275

[27] S. Beke , “A Review of the Growth of V_2_O_5_ Films from 1885 to 2010,” Thin Solid Films 519 (2011): 1761–1771.

[28] N. C. Joshi , H. K. Joshi , and P. Gururani , “An Updated Review on the Potential of V_2_O_5_‐Based Materials for Zinc‐Ion Batteries,” Ionics 31 (2025): 1–12.

[29] R. Jia , C. Yin , and J. Hu , “Fabrication of NiCo‐Doped V_2_O_5_ Nanospheres as Cathode for Enhanced Zn^2+^ Storage Performance,” Colloids and Surfaces A: Physicochemical and Engineering Aspects 727 (2025): 138186.

[30] K. Zhu and W. Yang , “Vanadium‐Based Cathodes for Aqueous Zinc‐Ion Batteries: Mechanisms, Challenges, and Strategies,” Accounts of Chemical Research 57 (2024): 2887–2900. 39279672 10.1021/acs.accounts.4c00484 PMC11669087

[31] G. Yang , Q. Li , K. Ma , C. Hong , and C. Wang , “The Degradation Mechanism of Vanadium Oxide‐Based Aqueous Zinc‐Ion Batteries,” Journal of Materials Chemistry A 8 (2020): 8084–8095.

[32] X. Wang , Z. Zhang , M. Huang , J. Feng , S. Xiong , and B. Xi , “ *In Situ* Electrochemically Activated Vanadium Oxide Cathode for Advanced Aqueous Zn‐Ion Batteries,” Nano Letters 22 (2022): 119–127. 34931840 10.1021/acs.nanolett.1c03409

[33] K. Chen and D. Xue , “Materials Chemistry toward Electrochemical Energy Storage,” Journal of Materials Chemistry A 4 (2016): 7522–7537.

[34] S. T. Senthilkumar , Y. Wang , and H. Huang , “Advances and Prospects of Fiber Supercapacitors,” Journal of Materials Chemistry A 3 (2015): 20863–20879.

[35] Y. Guo , H. Jiang , B. Liu , et al., “Better Engineering Layered Vanadium Oxides for Aqueous Zinc‐ion Batteries: Going beyond Widening the Interlayer Spacing,” SmartMat 5 (2024): e1231.

[36] R. Alcántara , P. Lavela , K. Edström , et al., “Metal‐Ion Intercalation Mechanisms in Vanadium Pentoxide and Its New Perspectives,” Nanomaterials 13 (2023): 3149. 38133046 10.3390/nano13243149 PMC10746094

[37] Y. Bai , Y. Qin , J. Hao , H. Zhang , and C. M. Li , “Advances and Perspectives of Ion‐Intercalated Vanadium Oxide Cathodes for High‐Performance Aqueous Zinc Ion Battery,” Advanced Functional Materials 34 (2024): 2310393.

[38] Y. Yang , Y. Tang , S. Liang , et al., “Transition Metal Ion‐Preintercalated V_2_O_5_ as High‐Performance Aqueous Zinc‐Ion Battery Cathode with Broad Temperature Adaptability,” Nano Energy 61 (2019): 617–625.

[39] T. Wu , K. Zhu , C. Qin , and K. Huang , “Unraveling the Role of Structural Water in Bilayer V_2_O_5_ during Zn^2+^ ‐Intercalation: Insights from DFT Calculations,” Journal of Materials Chemistry A 7 (2019): 5612–5620.

[40] J. Zhao , H. Ren , Q. Liang , et al., “High‐Performance Flexible Quasi‐Solid‐State Zinc‐Ion Batteries with Layer‐Expanded Vanadium Oxide Cathode and Zinc/Stainless Steel Mesh Composite Anode,” Nano Energy 62 (2019): 94–102.

[41] J. Li , Y. Li , J. Yao , B. Huang , J. Jiang , and J. Yang , “Effect of Heat Treatment on the Electrochemical Performance of V_2_O_5_·nH_2_O as a Cathode Material for Aqueous Rechargeable Zinc Ion Batteries,” Journal of Industrial and Engineering Chemistry 115 (2022): 554–560.

[42] Y. Gu , Y. Han , Z. Qin , D. Li , and L. Wang , “A Strategy to Control Crystal Water Content in Hydrated Vanadium Oxide Cathode for Promoting Aqueous Rechargeable Zinc‐Ion Batteries,” Journal of Alloys and Compounds 911 (2022): 165102.

[43] M. Du , F. Zhang , X. Zhang , et al., “Calcium Ion Pinned Vanadium Oxide Cathode for High‐Capacity and Long‐Life Aqueous Rechargeable Zinc‐Ion Batteries,” Science China Chemistry 63 (2020): 1767–1776.

[44] H. Geng , M. Cheng , B. Wang , Y. Yang , Y. Zhang , and C. C. Li , “Electronic Structure Regulation of Layered Vanadium Oxide via Interlayer Doping Strategy toward Superior High‐Rate and Low‐Temperature Zinc‐Ion Batteries,” Advanced Functional Materials 30 (2020): 1907684.

[45] H. Wang , M. Liang , H. Ma , et al., “Micro Fe‐Doped V_2_O_5_ as Cathode Material for Aqueous Zinc‐Ion Battery Application,” Journal of Energy Storage 101 (2024): 113785.

[46] K. Li , Y. Liu , and X. Wu , “Mn^2+^ Intercalation into Hydrated Vanadium Pentoxide Nanosheets for Highly Durable Zinc Ion Batteries,” ACS Applied Nano Materials 6 (2023): 12439–12446.

[47] L. E. Mo , Y. Peng , Y. Huang , et al., “Confined Mn Ions Enhance Charge Transport for High‐Performance Hydrated Vanadium Oxide Cathode,” ACS Sustainable Chemistry & Engineering 12 (2024): 8083–8090.

[48] L. Ma , N. Li , C. Long , et al., “Achieving Both High Voltage and High Capacity in Aqueous Zinc‐Ion Battery for Record High Energy Density,” Advanced Functional Materials 29 (2019): 1906142.

[49] D. Kundu , B. D. Adams , V. Duffort , S. H. Vajargah , and L. F. Nazar , “A High‐Capacity and Long‐Life Aqueous Rechargeable Zinc Battery Using a Metal Oxide Intercalation Cathode,” Nature Energy 1 (2016): 1–8.

[50] X. Guan , Q. Sun , C. Sun , et al., “Tremella‐Like Hydrated Vanadium Oxide Cathode with an Architectural Design Strategy toward Ultralong Lifespan Aqueous Zinc‐Ion Batteries,” ACS Applied Materials & Interfaces 13 (2021): 41688–41697. 34436858 10.1021/acsami.1c11560

[51] S. Islam , M. H. Alfaruqi , D. Y. Putro , et al., “K^+^ Intercalated V_2_O_5_ Nanorods with Exposed Facets as Advanced Cathodes for High Energy and High Rate Zinc‐Ion Batteries,” Journal of Materials Chemistry A 7 (2019): 20335–20347.

[52] G. Su , S. Chen , H. Dong , et al., “Tuning the Electronic Structure of Layered Vanadium Pentoxide by Pre‐Intercalation of Potassium Ions for Superior Room/Low‐Temperature Aqueous Zinc‐Ion Batteries,” Nanoscale 13 (2021): 2399–2407. 33491718 10.1039/d0nr07358j

[53] Y. Yang , Y. Tang , G. Fang , et al., “Li^+^ Intercalated V_2_O_5_ · *n*H_2_O with Enlarged Layer Spacing and Fast Ion Diffusion as an Aqueous Zinc‐Ion Battery Cathode,” Energy & Environmental Science 11 (2018): 3157–3162.

[54] Q. Li , X. Ye , H. Yu , et al., “Pre‐Potassiated Hydrated Vanadium Oxide as Cathode for Quasi‐Solid‐State Zinc‐Ion Battery,” Chinese Chemical Letters 33 (2022): 2663–2668.

[55] J. A. Chen , X. Hou , X. Wang , et al., “Bi‐Intercalated Vanadium Pentoxide Synthesized *via* Hydrogen Peroxide‐Induced Phase Transition for Highly Stable Cathode in Aqueous Zinc Ion Batteries,” Journal of Materials Chemistry A 12 (2024): 11322–11331.

[56] D. Sun , M. Zhang , W. Wan , Y. Cao , and H. Chai , “Modified Vanadium Oxide with Enhanced Diffusion Kinetic for High Rate Aqueous Zinc‐Ion Batteries,” Journal of Energy Storage 73 (2023): 109236.

[57] X. Jia , R. Tian , C. Liu , J. Zheng , M. Tian , and G. Cao , “Stability and Kinetics Enhancement of Hydrated Vanadium Oxide via Sodium‐Ion Pre‐Intercalation,” Materials Today Energy 28 (2022): 101063.

[58] M. Tian , C. Liu , J. Zheng , et al., “Structural Engineering of Hydrated Vanadium Oxide Cathode by K+ Incorporation for High‐Capacity and Long‐Cycling Aqueous Zinc Ion Batteries,” Energy Storage Materials 29 (2020): 9–16.

[59] Y. Qi , J. Huang , L. Yan , et al., “Towards High‐Performance Aqueous Zinc‐Ion Battery via Cesium Ion‐Intercalated Vanadium Oxide Nanorods,” Chemical Engineering Journal 442 (2022): 136349.

[60] C. Xia , J. Guo , P. Li , X. Zhang , and H. N. Alshareef , “Highly Stable Aqueous Zinc‐Ion Storage Using a Layered Calcium Vanadium Oxide Bronze Cathode,” Angewandte Chemie 130 (2018): 4007–4012. 10.1002/anie.201713291 29432667

[61] T. Zhou , X. Du , and G. Gao , “Revealing the Role of Calcium Ion Intercalation of Hydrated Vanadium Oxides for Aqueous Zinc‐Ion Batteries,” Journal of Energy Chemistry 95 (2024): 9–19.

[62] K. Zhu , T. Wu , W. van den Bergh , M. Stefik , and K. Huang , “Reversible Molecular and Ionic Storage Mechanisms in High‐Performance Zn_0.1_V_2_O_5_·*n*H_2_O Xerogel Cathode for Aqueous Zn‐Ion Batteries,” ACS Nano 15 (2021): 10678–10688. 34100590 10.1021/acsnano.1c03684

[63] D. Setiawan , H. Lee , H. H. Kwak , S. T. Hong , and M. S. Chae , “Bi‐Layered Calcium Vanadium Oxide as a Cathode Material for Wet Organic Electrolyte‐Based Rechargeable Zn‐Ion Batteries,” Journal of Energy Storage 72 (2023): 108497.

[64] X. Ren , H. Liu , N. Wang , et al., “Dual Pre‐Insertion Strategy to Achieve High‐Performance Vanadium Oxide toward Advanced Cylindrical Zinc Ion Batteries,” ACS Sustainable Chemistry & Engineering 11 (2023): 16965–16974.

[65] Z. Pang , B. Ding , J. Wang , et al., “Metal‐Ion‐Inserted Vanadium Oxide Nanoribbons as High‐Performance Cathodes for Aqueous Zinc‐Ion Batteries,” Chemical Engineering Journal 446 (2022): 136861.

[66] C. Liu , M. Tian , M. Wang , et al., “Catalyzing Zinc‐Ion Intercalation in Hydrated Vanadates for Aqueous Zinc‐Ion Batteries,” Journal of Materials Chemistry A 8 (2020): 7713–7723.

[67] X. Pang , S. Ji , P. Zhang , et al., “Interlayer Doping of Pseudocapacitive Hydrated Vanadium Oxide via Mn^2+^ for High‐Performance Aqueous Zinc‐Ion Battery,” Electrochimica Acta 441 (2023): 141810.

[68] Y. Zhang , L. Zhao , A. Chen , and J. Sun , “Cr^3+^ Pre‐Intercalated Hydrated Vanadium Oxide as an Excellent Performance Cathode for Aqueous Zinc‐Ion Batteries,” Fundamental Research 1 (2021): 418–424.

[69] Q. Li , T. Wei , K. Ma , G. Yang , and C. Wang , “Boosting the Cyclic Stability of Aqueous Zinc‐Ion Battery Based on Al‐Doped V_10_O_24_ ·12H_2_O Cathode Materials,” ACS Applied Materials & Interfaces 11 (2019): 20888–20894. 31117461 10.1021/acsami.9b05362

[70] B. Hu , X. Yang , D. Li , et al., “Rare Earth Metals Ion Intercalated Hydrated Vanadium Oxides for High‐Performance Aqueous Zinc‐Ion Batteries,” Ceramics International 50 (2024): 8421–8428.

[71] Z. Chen , H. Liu , S. Fan , et al., “Inhibition of Vanadium Cathode Dissolution in Zinc‐Ion Batteries on Thermodynamics and Kinetics by Guest Pre‐Intercalation,” Advanced Energy Materials 14 (2024): 2400977.

[72] F. Ming , H. Liang , Y. Lei , S. Kandambeth , M. Eddaoudi , and H. N. Alshareef , “Layered Mg* _x_ * V_2_O_5_·*n*H_2_O as Cathode Material for High‐Performance Aqueous Zinc Ion Batteries,” ACS Energy Letters 3 (2018): 2602–2609.

[73] Y. Tong , X. Li , S. Su , et al., “Hydrated Lithium Ions Intercalated V_2_O_5_ with Dual‐Ion Synergistic Insertion Mechanism for High‐Performance Aqueous Zinc‐Ion Batteries,” Journal of Colloid and Interface Science 606 (2022): 645–653. 34411832 10.1016/j.jcis.2021.08.051

[74] S. Li , L. Wang , L. Chen , Y. Li , G. Zu , and J. Wang , “Nanostructured Layered Vanadium Oxide Modified by Hydrated Manganese Ions for Boosting Zn^2+^ Storage,” ACS Applied Nano Materials 7 (2024): 15162–15170.

[75] S. Deng , Y. Jiang , D. Huang , et al., “Driving Intercalation Kinetic through Hydrated Na+ Insertion in V_2_O_5_ for High Rate Performance Aqueous Zinc Ion Batteries,” Journal of Alloys and Compounds 891 (2022): 161946.

[76] T. Hu , Z. Feng , Y. Zhang , et al., ““Double Guarantee Mechanism” of Ca^2+^ ‐Intercalation and rGO‐Integration Ensures Hydrated Vanadium Oxide with High Performance for Aqueous Zinc‐Ion Batteries,” Inorganic Chemistry Frontiers 8 (2021): 79–89.

[77] S. Luo , J. Cui , S. Liang , et al., “Graphene‐Supported Mg^2+^ Intercalated V_2_O_5_ Nanoribbons as Cathode for Aqueous Zinc‐Ion Batteries,” ACS Applied Nano Materials 7 (2024): 1655–1663.

[78] J. Sun , Y. Liu , H. Jiang , et al., “Mn^2+^ as the “spearhead” Preventing the Trap of Zn^2+^ in Layered Mn^2+^ Inserted Hydrated Vanadium Pentoxide Enables High Rate Capacity,” Journal of Colloid and Interface Science 602 (2021): 14–22. 34118601 10.1016/j.jcis.2021.05.163

[79] Z. Feng , Y. Zhang , X. Yu , Y. Yu , C. Huang , and C. Meng , “Aluminum‐Ion Intercalation and Reduced Graphene Oxide Wrapping Enable the Electrochemical Properties of Hydrated V_2_O_5_ for Zn‐Ion Storage,” Colloids and Surfaces A: Physicochemical and Engineering Aspects 641 (2022): 128473.

[80] W. Zhou , J. Chen , C. He , et al., “Hybridizing δ‐Type Na_x_V_2_O_5_·nH_2_O with Graphene towards High‐Performance Aqueous Zinc‐Ion Batteries,” Electrochimica Acta 321 (2019): 134689.

[81] X. Niu , J. Chen , and Y. Tan , “Graphene‐Assisted Improve Electrochemical Performance of Manganese Vanadium Oxide for Aqueous Zinc‐Ion Battery,” Next Energy 5 (2024): 100180.

[82] M. Zhou , J. Ma , W. Yang , et al., “Carbon‐coated Vanadium Oxide Nanoflowers with K^+^ Ions Pre‐embedment as a High‐rate Cathode for Zinc‐Ion Batteries,” ChemNanoMat 8 (2022): e202200047.

[83] J. Ren , P. Hong , Y. Ran , B. Wang , T. Chen , and Y. Wang , “High‐Loading and High‐Performance Zinc Ion Batteries Enabled by Electrochemical Conversion of Vanadium Oxide Cathodes,” Electrochimica Acta 415 (2022): 140265.

[84] C. Chen , B. Hou , T. Cheng , et al., “Sodium‐Intercalated Vanadium Oxide Coated on Carbon Cloth for Electrode Materials in High‐Performance Aqueous Zinc‐Ion Batteries,” Molecules 30 (2025): 2074. 40363878 10.3390/molecules30092074 PMC12073733

[85] D. Yan , Y. Li , J. Huo , R. Chen , L. Dai , and S. Wang , “Defect Chemistry of Nonprecious‐Metal Electrocatalysts for Oxygen Reactions,” Advanced Materials 29 (2017): 1606459. 10.1002/adma.201606459 28508469

[86] J. Li , Y. Li , W. Xu , Q. Huang , B. Liu , and J. Yao , “Preparation of Na+ Preintercalated V_2_O_5_·nH_2_O Nanobelts with Abundant Oxygen Vacancies as a High‐Performance Cathode Material for Aqueous Zinc‐Ion Batteries,” Journal of Alloys and Compounds 1003 (2024): 175646.

[87] H. Chen , J. Huang , S. Tian , et al., “Interlayer Modification of Pseudocapacitive Vanadium Oxide and Zn(H_2_O)_n_ ^2+^ Migration Regulation for Ultrahigh Rate and Durable Aqueous Zinc‐Ion Batteries,” Advancement of Science 8 (2021): 2004924. 10.1002/advs.202004924 PMC8292880 34029009

[88] B. Zhang , X. Han , W. Kang , and D. Sun , “Structure and Oxygen‐Defect Regulation of Hydrated Vanadium Oxide for Enhanced Zinc Ion Storage via Interlayer Doping Strategy,” Nano Research 16 (2023): 6094–6103.

[89] Y. Ba , G. Yang , S. Sun , et al., “K_0.39_V_2_O_5_·_0.52_H_2_O Nanostructures with Oxygen Vacancies as Cathodes for Aqueous Zinc‐Ion Batteries,” ACS Applied Nano Materials 8, 2025): 1205–1213.

[90] J. Gao , C. Cheng , L. Ding , G. Liu , T. Yan , and L. Zhang , “Synergistic Interlayer and Defect Engineering of Hydrated Vanadium Oxide toward Stable Zn‐Ion Batteries,” Chemical Engineering Journal 450 (2022): 138367.

[91] R. Liu , J. Zhang , C. Huang , et al., “Oxygen Defects Engineering and Structural Strengthening of Hydrated Vanadium Oxide Cathode by Coating Glucose Hydrothermal Carbon and Pre‐Embedding Mn (II) Ion for High‐Capacity Aqueous Zinc Ion Batteries,” Journal of Colloid and Interface Science 654 (2024): 279–288. 37844499 10.1016/j.jcis.2023.09.045

[92] Y. Jiang , J. Lu , W. Liu , et al., “Novel Polymer/Barium Intercalated Vanadium Pentoxide with Expanded Interlayer Spacing as High‐Rate and Durable Cathode for Aqueous Zinc‐Ion Batteries,” ACS Applied Materials & Interfaces 14 (2022): 17415–17425. 35389628 10.1021/acsami.2c01698

[93] Y. Tong , S. Su , X. Li , et al., “Synergistic Iron Ion and Alkylammonium Cation Intercalated Vanadium Oxide Cathode for Highly Efficient Aqueous Zinc Ion Battery,” Journal of Power Sources 528 (2022): 231226.

[94] L. Hu , Q. Sun , H. Cai , J. Ni , and J. Zhang , “Metal Ions and Organic Molecule Co‐Intercalated Vanadium Oxide Cathode for High‐Performance Zinc‐Ion Batteries,” Materials Science in Semiconductor Processing 177 (2024): 108358.

[95] Y. Yang , G. Liu , Z. Fang , et al., “Dual‐Guest Intercalated Vanadium Oxides with a Robust Channel for Efficient Zn Ion Diffusion and Storage,” ACS Sustainable Chemistry & Engineering 12 (2024): 3941–3950.

[96] F. Zhang , X. Sun , M. Du , et al., “Weaker Interactions in Zn^2+^ and Organic Ion‐pre‐intercalated Vanadium Oxide toward Highly Reversible Zinc‐ion Batteries,” Energy Environmental Materials 4 (2021): 620–630.

[97] K. Guo , Z. Song , Y. Lv , L. Gan , and M. Liu , “Inorganic–Organic Co‐Intercalated [Al_0.16_ (C_5_H_14_ON)_0.12_]V_2_O_5_·0.39H_2_O Cathode for High‐Performance Aqueous Zinc‐Ion Batteries,” Advanced Functional Materials 35 (2025): 2506036.

[98] P. Luo , G. Yu , W. Zhang , et al., ““Triple‐Synergistic Effect” of K^+^ and PANI Co‐Intercalation Enabling the High‐Rate Capability and Stability of V_2_O_5_ for Aqueous Zinc‐Ion Batteries,” Journal of Colloid and Interface Science 659 (2024): 267–275. 38176236 10.1016/j.jcis.2023.12.167

[99] Y. Zhang , Y. Du , B. Song , et al., “Manganese‐Ions and Polyaniline Co‐Intercalation into Vanadium Oxide for Stable Zinc‐Ion Batteries,” Journal of Power Sources 545 (2022): 231920.

[100] Q. Zong , Y. Zhuang , C. Liu , et al., “Dual Effects of Metal and Organic Ions Co‐Intercalation Boosting the Kinetics and Stability of Hydrated Vanadate Cathodes for Aqueous Zinc‐Ion Batteries,” Advanced Energy Materials 13 (2023): 2301480.

[101] D. Chen , X. Rui , Q. Zhang , et al., “Persistent Zinc‐Ion Storage in Mass‐Produced V2O5 Architectures,” Nano Energy 60 (2019): 171–178.

[102] L. Zhang , X. Qin , L. Wang , Z. Zhao , L. Mi , and Q. Lu , “Vanadium Oxide Cathode with Synergistic Engineering of Calcium‐Ion Intercalation and Polyaniline Coating for High Performance Zinc‐Ion Batteries,” Frontiers of Chemical Science and Engineering 17 (2023): 1244–1253.

[103] J. Liu , H. Hu , T. Yuan , P. Zhao , H. Liu , and H. Cheng , “Construction of Vanadium Oxide Cathode Material with High Performance and Stability and Its Application in Aqueous Zinc‐Ion Battery,” Applied Surface Science 648 (2024): 159005.

[104] S. Shen , Y. Li , Y. Dong , et al., “Vanadium Oxide Cathode Coinserted by Ni^2+^ and NH_4_ ^+^ for High‐Performance Aqueous Zinc‐Ion Batteries,” ACS Applied Materials & Interfaces 16 (2024): 8922–8929. 38330215 10.1021/acsami.3c18754

[105] M. Zhang , H. Wu , P. Chang , and L. Pan , “Hybrid Ternary Co‐Intercalation in the Interlayer of a Vanadium Oxide Cathode Enables High‐Capacity and Stable Zinc‐Ion Batteries,” Journal of Materials Chemistry A 13, 2025): 15702–15712.

[106] J. Qian , Y. You , Z. Fan , et al., “Mn Pre‐Intercalated Hydrated Vanadium Pentoxide Activated by Nitrogen Plasma for Enhanced Zinc Ion Storage,” Journal of Energy Storage 63 (2023): 106988.

[107] X. Zhang , R. Bian , Z. Sang , et al., “Anion and Cation Co‐Modified Vanadium Oxide for Cathode Material of Aqueous Zinc‐Ion Battery,” Batteries 9 (2023): 352.

[108] J. Chen , M. Shen , S. Song , et al., “Dual Ions Co‐Doped V_2_O_5_ as a Long‐Life and Stable Cathode for Aqueous Zinc‐Ion Batteries,” Materials Letters 387 (2025): 138262.

[109] W. Liu , X. Liu , F. Ning , et al., “Fabrication of a Heterovalent Dual‐Cation Pre‐Embedded Hydrated Vanadium Oxide Cathode for High‐Performance Zinc Ion Storage,” Journal of Materials Chemistry A 12 (2024): 11883–11894.

[110] Z. Feng , Y. Zhang , J. Sun , et al., “Dual Ions Enable Vanadium Oxide Hydration with Superior Zn²^+^ Storage for Aqueous Zinc‐Ion Batteries,” Chemical Engineering Journal 433 (2022): 133795.

[111] Z. Li , L. Yang , S. Wang , K. Zhu , and H. Li , “Co‐Insertion of K^+^ and Ca^2+^ in Vanadium Oxide as High‐Performance Aqueous Zinc‐Ion Battery Cathode Material,” Journal of Alloys and Compounds 992 (2024): 174589.

[112] S. Li , X. Jia , J. Liu , Z. Liu , and G. Cao , “Engineering Hydrated Vanadium Oxide by K^+^ and Ni^2+^ Incorporation for Aqueous Zinc Ion Batteries,” Materials Chemistry and Physics 287 (2022): 126358.

[113] L. Fan and Z. Li , “Highly Stable Cathode Materials for Aqueous Zn Ion Batteries: Synergistic Effect of Pre‐Inserted Bimetallic Ions in Vanadium Oxide Layer,” Journal of Alloys and Compounds 910 (2022): 164872.

[114] B. Hu , X. Yang , D. Li , et al., “Bimetallic Ions Pre‐Intercalated Hydrated Vanadium Oxides for High‐Performance Aqueous Zinc‐Ion Batteries,” Journal of Alloys and Compounds 1008 (2024): 176801.

[115] H. Jiang , Y. Zhang , M. Waqar , et al., “Anomalous Zn^2+^ Storage Behavior in Dual‐Ion‐In‐Sequence Reconstructed Vanadium Oxides,” Advanced Functional Materials 33 (2023): 2213127.

[116] M. Y. Ma , Y. Liu , J. L. Yang , et al., “Multi‐Metal Ions Co‐Regulated Vanadium Oxide Cathode toward Long‐Life Aqueous Zinc‐Ion Batteries,” Journal of Colloid and Interface Science 670 (2024): 174–181. 38761570 10.1016/j.jcis.2024.05.065

[117] A. N. Guerreiro , I. B. Costa , A. B. Vale , and M. H. Braga , “Distinctive Electric Properties of Group 14 Oxides: SiO_2_, SiO, and SnO_2_ ,” International Journal of Molecular Sciences 24 (2023): 15985. 37958967 10.3390/ijms242115985 PMC10649876

[118] A. N. Guerreiro , M. C. Baptista , B. A. Maia , and M. H. Braga , “Interfacial Chemistry with ZnO: *In* *Operando* Work Functions in Heterocells,” ACS Applied Energy Materials 5 (2022): 9811–9822.

[119] A. N. Guerreiro , B. A. Maia , H. Khalifa , M. C. Baptista , and M. H. Braga , “What Differentiates Dielectric Oxides and Solid Electrolytes on the Pathway toward More Efficient Energy Storage?,” Batteries 8 (2022): 232.

[120] Y. Zhang , S. Gao , Q. Li , et al., “Bifunctional Organic Molecule Co‐Intercalated Aluminum Vanadate for Highly Reversible Aqueous Zinc‐Ion Batteries,” Advanced Functional Materials 36 (2026): e22432.

[121] S. Chen , K. Li , K. S. Hui , and J. Zhang , “Regulation of Lamellar Structure of Vanadium Oxide via Polyaniline Intercalation for High‐Performance Aqueous Zinc‐Ion Battery,” Advanced Functional Materials 30 (2020): 2003890.

[122] H. Song , Y.‐F. Cui , Y. Li , et al., “Alcohol Molecule Coupling: A Universal Approach to Modulating Amorphousness in Vanadium‐Based Cathodes for High‐Rate and Durable Aqueous Zinc‐Ion Batteries,” Science Advances 11 (2025): eadt7502. 40408486 10.1126/sciadv.adt7502 PMC12101504

[123] J. Liu , W. Shang , S. Chen , W. Cai , X. Guan , and J. Zhang , “Organic Intercalation‐Induced Interlayer Engineering of V_2_CT_X_ MXene for High‐Performance Zinc‐Ion Batteries,” Chemical Engineering Journal 521 (2025): 166812.

